# Factors Influencing Isolation Behavior of Dogs: A Holder-Based Questionnaire and Behavioral and Saliva Cortisol Responses during Separation

**DOI:** 10.3390/ani13233735

**Published:** 2023-12-02

**Authors:** Jennifer Silbermann, Udo Gansloßer

**Affiliations:** 1 Institute of Ecology and Evolution, Friedrich Schiller University Jena, Dornburger Str. 159, 07743 Jena, Germany; 2 Institute of Zoology and Evolutionary Research with Phyletischem Museum, Ernst-Haeckel-Haus and Biodidactics, Friedrich Schiller University Jena, Erbertstr. 1, 07743 Jena, Germany; udo@ganslosser.de

**Keywords:** dog, separation, behavior, cortisol, video recordings, questionnaire study, welfare

## Abstract

**Simple Summary:**

Separation problems in dogs are common and can manifest in symptoms such as restlessness, destruction, or vocalization. This study examines how separation behavior differs between dogs with and without separation problems and the possible risk factors. An online questionnaire with 940 participants was performed. After two groups were formed, depending on whether the holders stated that the dogs showed typical symptoms during separation, the groups were examined for differences. Furthermore, a separation test with videotaping and cortisol sampling of six dogs used to being separated but without separation problems was carried out. It was found that separation problems were primarily characterized by physical activity and vocalization. Dogs with separation-related problems needed more time to relax after separation. Dogs that were greeted after separation were less likely to have separation problems than dogs that were less calm and more pessimistic, excited and persistent. During the test it was found that dogs without separation problems were mostly inactive. Understanding these differences may help to diagnose separation-related problems and modify or even avoid risk factors to improve animal welfare.

**Abstract:**

This study examined how separation behavior differs between dogs with and without separation-related problem behavior (SRB) and the possible risk factors. The study consisted of an online survey with 940 dog holders, which, in addition to demographic facts, also includes personality, emotional disposition and the attachment by the holder. Furthermore, a separation test was carried out with six non-SRB dogs over a maximum of 6 h, in which behavior and cortisol were determined. The questionnaire revealed that SRB dogs differed significantly from non-SRB dogs regarding the following factors: symptoms with at least a medium effect size such as restlessness, excitement, whining, howling, lip licking, barking and salivation, time to relax after separation, pessimism, persistence, excitability, calmness, separation frequency, greeting of holder and type of greeting. There were several other differences, but with weak effect sizes. The test showed that non-SRB dogs were mostly inactive during separation (lying resting and lying alert). Vocalization was almost non-existent. Behavior and cortisol did not change significantly over the different time periods. The data demonstrated typical symptoms and possible risk factors, some of which may be avoided or changed to improve animal welfare.

## 1. Introduction

Dogs that live close to humans share their living space and everyday life with each other. In industrialized countries, it is common for people to work 6 to 8 h a day. Dogs are inevitably affected by being separated from their holders [1,2,3,4]. Various authors have reported that separation issues are one of the most common behavior problems of dogs and that many dogs end up in shelters, or, depending on the country, are euthanized. The question of what influences separation behavior and separation problems is therefore not only a scientific question, but also an important one in terms of animal welfare.

In some studies, it could be shown that the behavior of dogs without separation problems during the separation from the holder consisted mostly of resting behavior and their activity levels were very low [1,2,5,6]. Rehn and Keeling [2] videotaped dogs and recorded heart rates while separated from holders for 0.5, 2, or 4 h. The dogs had no separation problems and spent most of the time resting. The behavior during the separation did not differ with increasing duration, but the greeting behavior after reunion was different. After a longer separation, the dogs showed a more intense greeting, increased physical activity and more attention towards the holder [2]. Researchers hypothesized that dogs may therefore perceive duration in some way and that behavior may not be exhibited during separation because the costs would outweigh the benefits [2]. The type of greeting after a separation seems to have importance in assessing separation behavior.

A study by Rehn et al. [7] showed that the holder greeting influences cortisol and oxytocin levels in dogs. It was found that holders who greeted their dog verbally and physically caused significantly higher increases in oxytocin in the dog than those who greeted them verbally or not at all. Oxytocin, in turn, was able to compensate for cortisol [7]. Thus, not only does the greeting itself have an effect, but the type of greeting matters. The study suggests that it makes sense to observe the greeting situation between dog and holder more closely and to consider the type of greeting.

According to Konok et al. [1], dogs without separation problems behave differently during greetings than dogs with separation problems. After a separation from the holder, dogs without separation problems showed less stress-related behavior, sought closeness to the holder more often and were less active. Dogs with separation problems, on the other hand, were very active, but rarely sought the proximity of the holder and instead moved through the room [1]. Another interesting discovery by Konok et al. [1] was that dogs with separation problems could not simply be calmed by the holder. Therefore, this result could suggest that the time to relaxation after the separation could be a good predictor of the presence of separation problems.

When dogs have separation problems, holders report that they mostly vocalize, destroy and are very physically active during separation [8]. Konok et al. [1] found that activity in dogs with separation problems did not decrease over the duration of the separation, in contrast to dogs without separation problems. In a study by Palestrini et al. [9], it was observed that while dogs spent most of their time on vocalization, environmental orientation, panting, passive behavior and destruction, barking and orientation decreased over time, but panting was more common as time went by.

Vocalization is often cited as one of the most common symptoms of separation problems [8,9,10]. Pongracz et al. [11] dealt with which type of vocalization plays a role in separation anxiety. To this end, they examined the frequency of howling, barking and whining during a separation in dogs with and without separation anxiety. It was found that dogs with separation anxiety whined significantly more often, but the dogs did not differ in levels of barking. The researchers suspected that the types of vocalizations occurred depending on the emotional state during the separation. Whining was associated with fear and barking with frustration [11].

The causes of separation problems are not well known, but there is ample evidence that attachment, personality traits and emotional state may be important. Mendl et al. [12] used a test to determine how pessimistic dogs were. It was then shown that the probability of showing separation problems increased with higher pessimism scores. Emotional disposition therefore appears to be important when considering separation behaviors in dogs. Pessimism aside, Konok et al. [13] showed that dogs with high levels of neuroticism tended to have separation problems more often.

Some researchers believe that so-called hyperattachment causes separation problems [14,15]. Other studies have shown that there is a connection to the attachment style [13,16]. When holders scored high on attachment avoidance, they were more likely to have dogs with separation problems. The researchers suspected that, analogous to the mother–child bond, holders with an avoidant attachment style were less responsive, and are therefore not a secure base, resulting in an insecure bond between the dog and the holder. The follow-up study by Konok et al. [16] confirmed these results because holders who were less worried about their dog during separation were more likely to have dogs with separation problems.

In addition to all these relations to separation behavior, there are other circumstances that have shown that they are positively related to the occurrence of separation problems [8,11,14,17,18,19,20,21,22,23,24,25,26,27]. These are specified in more detail in the Methods section.

The available findings give rise to different questions for this work. On the one hand, the non-pathological separation behavior of dogs should be examined more closely. For this purpose, a test was carried out that included a separation of up to 6 h, since a maximum test duration of 4 h is known from the literature [2]. Behavior was recorded during separation, but changes in cortisol level were also noted, since cortisol is said to provide information about the dog’s physiological stress and to the authors knowledge, there is no study to date that tested dogs for 6 h and included cortisol measurements. In addition, by using a questionnaire, an attempt was made to determine connections between the separation behavior and risk factors known from the literature, but also the personality and emotional disposition of the dog, as well as the attachment of the holder.

We hypothesized that dogs with and without separation-related problems would differ regarding the risk factors known from the literature, but also show differences regarding their personality, emotional disposition and the attachment of the holder [12,13,16]. Bearing in mind the findings of previous studies, we assumed that dogs without separation problems were primarily inactive during the test and that this would not change if separated for around 6 h [1,2,5,6].

The overall goal of this work was to better understand how non-pathological separation behavior differs from separation problems and which factors affect separation. This knowledge could help to get a little closer to diagnosing separation problems and identify possible risk factors that may be modified or even avoided so that our dogs have fewer separation problems, and their welfare would be improved.

## 2. Material and Methods

The study consisted of two parts. Non-pathological separation behavior was assessed in a test in which the separation from the holder was videotaped and cortisol testing took place. An online survey was carried out to examine behavior in the presence of separation problems and to determine possible influencing factors.

### 2.1. Test

Participants were found by distributing digital flyers with information about the project and a link in Facebook groups on various dog topics. The participants were asked to fill in the questionnaire via Google Forms. After that, the authors selected participants based on the specified requirements.

To participate in the test, there were several requirements that had to be met. It was important that the participating dogs were used to the tested separation durations. Therefore, only dogs that were familiar with a minimum of 6 h of separation were selected. Additionally, single-dog households were chosen. The participation only took place if the dog did not show any obvious separation-related behavior problems.

#### 2.1.1. Video Analysis

**Figure 1:**
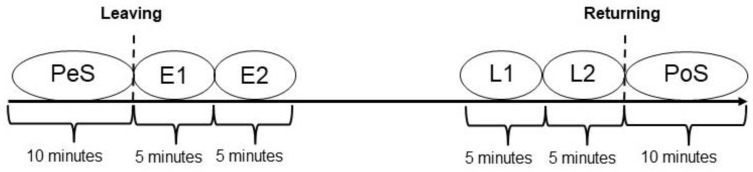
Test design adapted from Rehn and Keeling [2].

Before the test began, the participants were given written instructions (Figure S1). Dogs were videotaped for approximately 2, 4 and 6 h. The recording started at least 10 min before leaving and ended at least 10 min after the return of the holder. To compare the different separation durations, six intervals were analyzed. This included a 10 min interval before leaving and after returning, hereinafter referred to as pre-separation (PeS) and post-separation (PoS), and four 5 min intervals that were evaluated during the separation. Two intervals began immediately after leaving, referred to as Early 1 (E1) and Early 2 (E2), and two of them started before the holder returned, referred to as Late 1 (L1) and Late 2 (L2) (Figure 1). The test design was based on the study by Rehn and Keeling [2] to achieve better comparability. Focal animal sampling according to Altmann [28] was used.

All coded behaviors and their definitions are shown in Table 1. In addition to the individual behaviors, seven behavior groups were formed: physical activity, attentive behavior, interaction by holder, interaction by dog, vocalizing, stress behavior and restlessness. Transitions between standing, walking, lying, sitting, and running were defined as restlessness. The recorded behaviors and the behavior groups were adapted from Rehn and Keeling [2]. All behaviors were coded every 15 s as present or absent during all six intervals. For coding, the software Solomon Coder beta 19.08.02 was used [29]. After coding, all data were transferred into Microsoft Excel.

#### 2.1.2. Cortisol

To cause as little stress as possible through the procedure of saliva sampling and to build up a positive expectation that generates an increased flow of saliva, the dogs were trained prior to testing (Figure S2). For collecting the saliva, the Salivette^®^ Cortisol by Sarstedt (Sarstedt AG & Co. KG, Nümbrecht, Germany) was used. All important test data were recorded in the protocol (Figure S3).

Cortisol was measured for two periods: after approximately 2 and 6 h of separation from the holder. Samples were taken on the test day in the morning after waking up, before and after the test. The morning sample was taken between 7 and 10 a.m. The dog should not have eaten, drunk or exercised before, as this can affect cortisol levels. The second saliva sample was taken before the holder got ready to leave. The dog should not have eaten or drunk for at least 30 min and not exercised intensively for 60 min. After the separation, the sample was taken after the holder greeted the dog. It was pointed out that the collection should not last longer than 4 min, as Kobelt et al. [30] noticed that otherwise the cortisol level could be influenced by the sampling. However, if the sampling was prolonged, this was noted on the protocol. All samples were stored in a freezer as soon as possible after testing.

After the last sample collection, samples were sent in the post and were frozen again until the samples of all participants were sent to the laboratory (Prof. Dr. C. Kirschbaum, Biopsychology—TU Dresden, Dresden, Germany) as a frozen express shipment. There are no significant effects of varying temperature and repeated freezing and thawing (up to four times) on the cortisol level in saliva samples [31,32,33].

Until analysis, saliva samples were frozen at −20 °C. For analysis the samples were thawed and afterwards centrifuged at 3000 rpm for 5 min. To measure salivary cortisol concentrations an immunoassay (ELISA) with high sensitivity (IBL International, Hamburg, Germany) were used and intra- and interassay coefficients were below 8%.

### 2.2. Online Survey

The acquisition of participants was the same as in the test, but care was taken to distribute the flyer to other groups.

#### 2.2.1. Creation of Separation Questionnaire

The survey consisted of four different questionnaires, one of which was created by the authors and is referred to as separation questionnaire.

Before the separation questionnaire was created, the current literature was researched to find out which factors could have a possible influence on the separation behavior (Table S1).

In addition, behaviors during separation that, according to studies, can indicate separation problems were part of the questionnaire [9,14,15,16,17,21,34,35]. The presence and frequency of the following behaviors during separation were asked about: howling, whining, barking, growling, salivation, urination, defecation, destruction, restlessness, shaking, excitement, yawning, lip licking and escape behavior. The holders were asked to indicate the behavior according to the frequency of occurrence on a scale from one (never) to four (always). The rating of the individual behaviors resulted in a total separation score. If at least one of the behaviors was rated a three or four, separation-related behavior was assumed to be exhibited and the dog was counted as separation-related behavior (SRB) dog. The final structure of the questionnaire is shown in Table S2.

Before the separation questionnaire was distributed online, a pre-test was carried out in which 15 people took part. Suggestions for improvement submitted by the participants for understanding or a more sensible selection were evaluated and partially implemented.

#### 2.2.2. Used Questionnaires

The last part of the questioning consisted of three questionnaires that have already been validated and covered the following areas: attachment, personality and emotional disposition.

The questionnaire dealing with the emotional attachment of the holder to the pet is called Lexington Attachment to Pet Scale (LAPS). It was created by Johnson et al. [36], translated into German by Hielscher et al. [37] and consists of 23 questions. The questions deal with various topics and are summarized as people substituting, animal rights and welfare, and general attachment. Every question must be answered with a rating between zero (strongly disagree) and three (strongly agree).

The questionnaire developed by Turcsán et al. [38] was used to record the personality of the dogs and consists of 24 questions. There are 17 items belonging to four categories: boldness, trainability, dog sociability and calmness [38]. In the questionnaire by Turcsán et al., “boldness” does not correspond to bold, which is a supertrait in the shy–bold concept, but is to be understood as extraversion [38]. In the following, it is therefore called extraversion. The participants were asked to rate their dog by using a 3-point scale between zero (strongly disagree) and two (strongly agree).

Sheppard and Mills [39] developed a psychometric scale to make statements about a dog’s emotional predisposition. The final questionnaire consists of 21 questions and was translated by Udo Gansloßer. The questions are answered by using a 5-point Likert scale between one (strongly disagree) and five (strongly agree). By summing up, scores of different categories are the result: pessimism, interest, persistence and excitement. Finally, pessimism, negative activation and positive activation can be compared by summing up interest, persistence and excitement to form the category positive activation.

### 2.3. Statistical Analysis

The questionnaire data were exported to Excel and cleaned up. Duplicates were found and removed. If answers to questions were not clear, they were treated as missing data for that cell. In the absence of responses to questions on the symptoms, attachment, personality and emotional disposition, no score was formed for that dog and it was treated as missing data. For open-ended questions, answers were sorted into categories. Further statistical evaluations and the creation of diagrams in R Studio then took place. Subsequently, the validation of the SRB status was established, in which SRB and non-SRB dogs were checked for differences using Welch’s *t*-test. All variables were then reviewed for their impact on SRB status. If the data were nominal, a Chi-square test was used; if the cell size was less than five, the Fisher’s exact test was used. Mann–Whitney U test was applied for ordinal data and Welch’s test for metric data. Possible connections to separation duration and frequency, greeting intensity of dog and time to relaxation were examined using correlations according to Kendall’s τ, incorporating adjustment according to Holm–Bonferroni. Effect sizes were calculated using Cramér’s V for Chi-square and Fisher’s exact test, Pearson’s r for Mann–Whitney U test, and Cohen’s d for Welch’s *t*-test. *p* values < 0.05 were considered significant.

The differences between the cortisol levels at different time points were tested using an ANOVA and a *t*-test. Possible influences on the cortisol concentration were examined for nominal data using Mann–Whitney U test and for metric data using a correlation according to Kendall’s τ. For the video data, differences between separation classes were determined using Friedman tests. Separation classes were formed as categories for the duration of separation because videos were not exactly 2, 4 or 6 h long. Relationships between behaviors were made by multiple correlations according to Kendall’s τ with Holm–Bonferroni correction. Multiple correlations according to Kendall’s τ with Holm–Bonferroni correction were applied to evaluate connections between behavior and personality, attachment and emotional predisposition. Cortisol and behavior were also tested for correlation using Kendall’s τ and corrected with Holm–Bonferroni. *p* values < 0.05 were considered significant.

## 3. Results

### 3.1. Test

#### 3.1.1. Separation Behaviors

Six dogs took part in the test.

Separation class 1 represented an average separation time of 2:05:20 h, in separation class 2 it was 4:07:54 and in the third class it was 6:10:25 h.

It was found that the dogs spent most of their time lying resting (median = 42.73%, IQR = 30.63%, 71.52%) and lying alert (median = 21.48%, IQR = 15.39%, 38.13%). In 18.8% (IQR = 8.4%, 25.63%) of the time analyzed, attention was directed towards the holder. More than 10% of the time was also spent standing (median = 10%, IQR = 4.03%, 16.54%) or walking (median = 5.04%, IQR = 2.65%, 9.38%). The dogs spent less than 5% of the time with, in descending order: physical contact of holder (median = 3.36%, IQR = 0.63%, 7.05%), following holder (median = 1.32%, IQR = 0.66%, 1.92%), grooming (median = 0.83%, IQR = 0%, 4.64%), tail wagging (median = 0.66%, IQR = 0%, 2.01%), verbal contact with holder (median = 0.64%, IQR = 0%, 1.5%) and physical contact of dog (median = 0.62%, IQR = 0%, 2.69%) (Tables S3 and S4). Some behaviors did not occur at all: running, play, barking, growling, howling, lip licking, inviting play and holder invites play.

#### 3.1.2. Possible Influences on Separation Behavior

To evaluate whether the duration of the separation had an impact on behavior, the percentage in the interval between all separation classes was compared by applying Friedman tests (Table S5).

**Figure 2:**
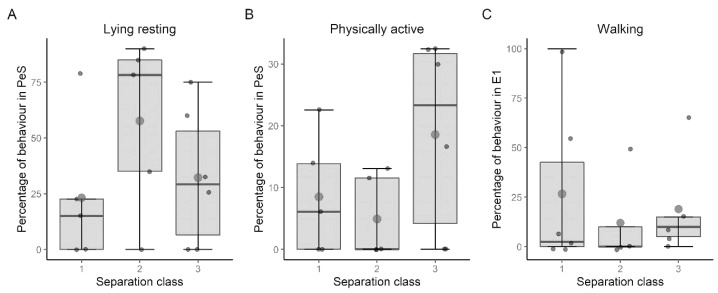
Differences among separation classes while (**A**) lying resting in interval PeS, (Sep. class 1: n = 5; Sep. class 2: n = 5; Sep. class 3: n = 6), (**B**) being physically active in interval PeS (Sep. class 1: n = 5; Sep. class 2: n = 5; Sep. class 3: n = 6) and (**C**) walking in interval E1 (Sep. class 1: n = 6; Sep. class 2: n = 5; Sep. class 3: n = 5).

In the period before separation occurred (PeS), there were significant differences in lying resting (χ^2^ (2, *N* = 16) = 6.5, *p* = 0.039) and physically active (χ^2^ (2, *N* = 16) = 6.0, *p* = 0.050). During the shortest separation period, a median of 15% of lying resting was observed, after approximately 4 h of separation this was 78.26% and after approximately 6 h this was 29.15% (Figure 2A). A post hoc analysis with Wilcoxon signed-rank tests was conducted with a Holm–Bonferroni correction applied, resulting in no significant differences (Sep. class 1 + 2: Z = 15, *p* = 0.188; Sep. class 1 + 3: Z = 10, *p* = 1; Sep. class 2 + 3: Z = 5, *p* = 1). The most physical activity was shown in separation class 3, after the longest separation, with a median of 23.33% (Sep. class 1: 6.06%; Sep. class 2: 0%) (Figure 2B). The post hoc test did not reveal any significant differences between the different separation durations (Sep. class 1 + 2: Z = 6, *p* = 0.543; Sep. class 1 + 3: Z = 0, *p* = 0.543; Sep. class 2 + 3: Z = 0, *p* = 0.543).

During the first minutes of the separation (E1), there were only different characteristics in the separation classes concerning walking (χ^2^ (2, *N* = 16) = 6.86, *p* = 0.032). While the median in separation class 1 was 2.5%, the behavior did not occur at all in separation class 2 and was 10% in separation class 3 (Figure 2C). It was found that none of the separation classes differed significantly from each other (Sep. class 1 + 2: Z = 2, *p* = 1; Sep. class 1 + 3: Z = 0, *p* = 0.292; Sep. class 2 + 3: Z = 0, *p* = 0.292).

No significant differences were found in all other behaviors.

**Figure 3:**
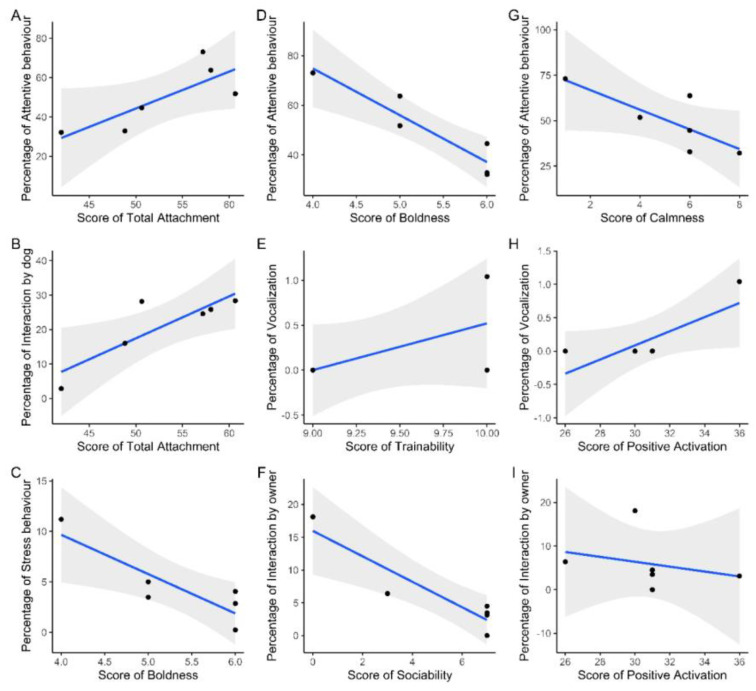
(**A**) Attentive + attachment (n = 6), (**B**) interaction (ia) dog + attachment (n = 6), (**C**) stress + boldness (n = 6), (**D**) attentive + boldness (n = 6), (**E**) vocalization + trainability (n = 6), (**F**) ia holder + dog sociability (n = 6), (**G**) attentive + calmness (n = 6), (**H**) vocalization + pos. act. (n = 6), (**I**) ia holder + pos. (n = 6).

To find out whether attachment, personality and emotional predisposition could relate to behavior during separation, the percentages of all behavior groups were correlated across all intervals. It was again corrected according to Holm–Bonferroni. There were no significant effects. However, the total attachment showed a strong effect with attentive behavior (τb = 0.60) and interaction by dog (τb = 0.73) (Figure 3A,B). For extraversion, there were strong correlations with stress behavior (τb = −0.70) and attentive behavior (τb = −0.86) (Figure 3C,D). With increasing trainability, the proportion of vocalization also increased (τb = 0.63) (Figure 3E). There was also a strong negative correlation between dog sociability and interaction by holder (τb = −0.77), and a strong effect of calmness with attentive behavior (τb = −0.75) (Figure 3F,G). The more points a dog scored for positive activation, the more it displayed vocalization (τb = 0.65) and the less the holder interacted with the dog (τb = −0.60) (Figure 3H,I).

#### 3.1.3. Cortisol

It was examined whether the cortisol difference before (difference in cortisol concentration before separation and morning concentration) differed from the cortisol difference after (difference in cortisol concentration after and before separation) separation.

**Figure 4:**
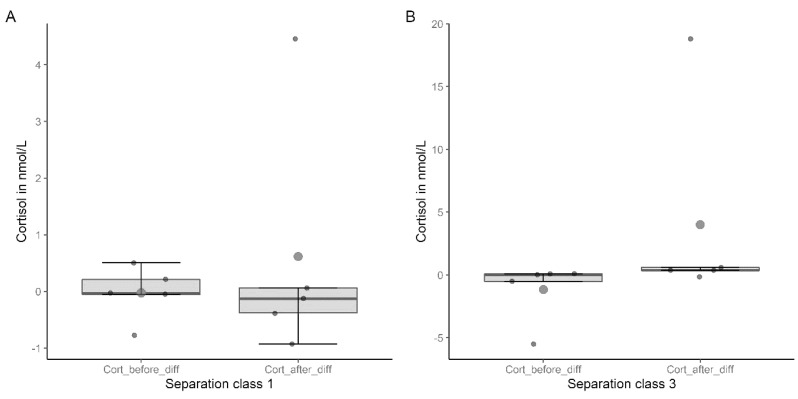
Differences in cortisol levels before and after separation in nmol/L in (**A**) separation class 1 (n = 5) and (**B**) separation class 3 (n = 5).

Although the cortisol difference before was negative for both separation durations (Sep. class 1: −0.03 ± 0.48 nmol/L; Sep. class 3: −1.16 ± 2.44 nmol/L) and positive for cortisol difference after (Sep. class 1: 0.62 ± 2.18 nmol/L; Sep. class 3: 4.00 ± 8.28 nmol/L), no significant difference was found (Sep. class 1: t = −0.55, *p* = 0.61; Sep. class 3: t = −1.08, *p* = 0.34) (Figure 4A,B).

Multiple tests were applied to examine cortisol changes across different durations of separation, but no significant effect could be observed.

### 3.2. Online Survey

There were 940 valid answers from participants in the questionnaire. Since not all questions had to be answered or the answers were not always clear, there were fewer than 940 for some variables.

#### 3.2.1. Symptoms of Separation-Related Behavior Problems

It was found that 7.91% (67/847) of the participants had SRB dogs. To ensure that this procedure also represented the frequency of symptoms and the separation score, differences in these variables between the two groups were then tested. The separation score differed significantly (tWelch (67.29) = −11.16, *p* < 0.001, d = 3.20). On average, SRB dogs had a nearly seven-times higher score (M = 12.6, SD = 7.83) than non-SRB dogs (M = 1.86, SD = 2.64). Dogs belonging to the SRB group showed a mean of 5.33 (SD = 2.74) symptoms, showing a significant difference from the other dogs (tWelch (71.45) = −11.34, *p* < 0.001, d = 1.97), which had 1.45 (SD = 1.89) symptoms.

**Figure 5:**
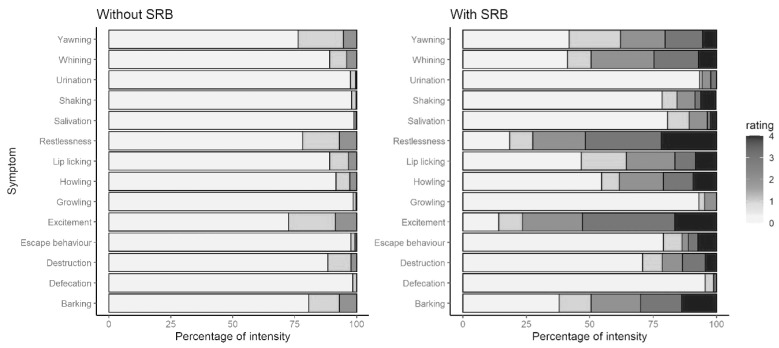
Differences between non-SRB and SRB dogs concerning distribution of symptoms during separation (non-SRB: n = 780; SRB: urination, defecation, destruction: n = 89; barking, restlessness: n = 87; howling, growling: n = 86; whining, shaking, excitement: n = 85; salivation: n = 83; escape behavior: n = 81; yawning: n = 74; lip licking: n = 73).

SRB dogs showed all symptoms significantly more frequently (Figure 5). The symptoms that achieved a *p* value of <0.001 (Mann–Whitney U-test) and at least a medium effect size were restlessness (r = 0.486), excitement (r = 0.473), whining (r = 0.426), howling (r = 0.363), lip licking (r = 0.357), barking (r = 0.350) and salivation (r = 0.328). Significant symptoms with a small effect size included shaking (r = 0.297), escape behavior (r = 0.280), yawning (r = 0.261), destruction (r = 0.174) and growling (r = 0.121).

#### 3.2.2. Possible Influences on Separation-Related Behavior Problems

All effect sizes and *p* values related to the SRB status are shown in Table S6.

With a significant difference, more SRB dogs (50.56%, n = 869) were mixed breeds (χ^2^ (1, *N* = 860) = 8.056, *p* = 0.005). In the non-SRB group, just 33.97% were not purebred. Cramer‘s phi was very weak (φ = 0.097).

The holders who had SRB dogs obtained their dogs less often (SRB: 40.45%; non-SRB: 57.44%) from breeders and more often from national and international animal shelters (SRB: 43.82%; non-SRB: 27.18%). The two groups differed significantly regarding the source of adoption (Fisher’s exact test: *p* = 0.017), but the effect was small with φ = 0.131.

Among the dogs without SRB, there were significantly fewer (n = 869, 28.59%) who had already been in an animal shelter than in the SRB group, where almost every second dog (46.07%) had experienced a shelter (χ^2^ (1, *N* = 869) = 10.726, *p* = 0.001, φ = 0.111).

In the SRB group, significantly more dogs (SRB: 47.19%, n = 89; non-SRB: 34.36%, n = 780) lived with another animal (χ^2^ (1, *N* = 869) = 5.187, *p* = 0.023). In both groups, the dogs mainly had one other dog with them (SRB: 49.44%; non-SRB: 48.72%), but significantly more SRB dogs (34.83%) were living together with a cat (χ^2^ (1, *N* = 857) = 4.033, *p* = 0.045).

**Figure 6:**
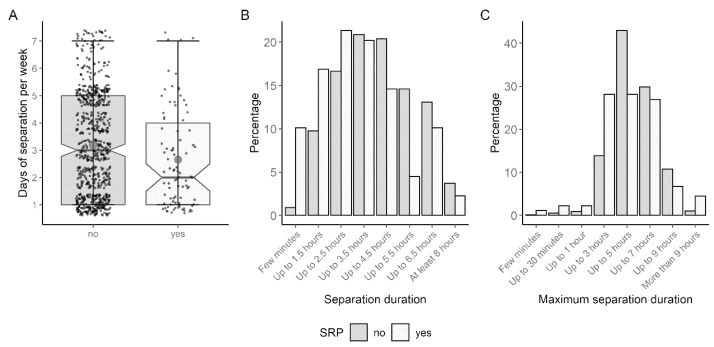
Group differences concerning (**A**) separation frequency per week (n = 869), (**B**) separation duration (n = 869) and (**C**) maximum separation duration (n = 869).

Dogs assigned to the SRB group were separated from their holders for an average of 2.65 days (SD = 1.75) a week, which was less frequently than dogs of the other group, which were separated for 3.18 days (SD = 1.84) (Figure 6A). With a *p* value of 0.009, this difference was significant (tWelch (111.56) = 2.674), but only a weak effect size (d = 0.287) was observed.

The distribution of both groups regarding the separation time differed significantly (U (n1 = 780, n2 = 89) = 43,459, z = −3.955, *p* < 0.001, r = 0.134). With a median of 3.5 h, SRB dogs were not left alone for as long as non-SRB dogs (median = 4.5 h) (Figure 6B). With a weak effect (r = 0.111), there was also a clear difference between the two groups in terms of the maximum length of time the holders were willing to leave their dog alone (U (n1 = 780, n2 = 89) = 41,649, z = −3.263, *p* = 0.001) (Figure 6C). Most SRB holders would leave their dogs alone for a maximum of 3 to 5 h (28.09% each), while 13.85% chose a maximum separation time of 3 h and 42.95% chose a maximum of 5 h. More holders of SRB dogs would leave them for more than 9 h (SRB: 4.49%; non-SRB: 1.03%).

**Figure 7:**
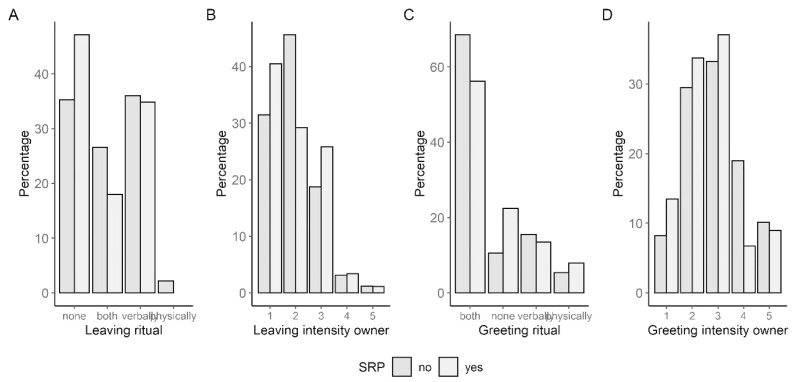
Group differences concerning (**A**) type of leaving ritual (n = 869), (**B**) leaving intensity of holder (n = 869), (**C**) type of greeting ritual (n = 869) and (**D**) greeting intensity of holder (n = 869).

Whether the holder had a leaving ritual with the dog was affirmed by more than half (52.81%, n = 89) of the SRB group and almost two-thirds (64.74%, n = 780) of the other group, with a significant difference (χ^2^ (1, *N* = 869) = 4.409, *p* = 0.036, φ = 0.071). The type of leaving ritual was almost similarly distributed in both groups (Fisher’s exact test: *p* = 0.071) (Figure 7A). The difference between the groups was obvious in the fact that more SRB dog holders did not say goodbye to their dogs at all (SRB: 47.19%; non-SRB: 35.26%) and more non-SRB dog holders said goodbye to their dogs verbally and physically (SRB: 17.98%; non-SRB: 26.53%).

Concerning the location during the separation, there were several differences between the two groups. With a *p* value of 0.007, more non-SRB dogs lived inside (SRB: 98.97%, n = 780; non-SRB: 94.38%, n = 89) but the effect was negligible (φ = 0.099). A total of 33.71% ((n = 89) of SRB dogs stayed in only one room during the separation, which was significantly more frequently (χ^2^ (1, *N* = 869) = 9.761, *p* = 0.002, φ = 0.106) than the other group (18.97%, n = 780). More rarely than non-SRB dogs, SRB dogs had several rooms available (SRB: 52.81%; non-SRB: 76.54%). The difference was significant but a weak effect size of φ = 0.160 was calculated (χ^2^ (1, *N* = 869) = 22.223, *p* < 0.001). While 8.99% (n = 89) of the SRB dogs were in an inside kennel, which is legally prohibited in Germany, there were significantly fewer in the group without SRB, with 3.21% kept inside (n = 780) (Fisher’s exact test: *p* = 0.014, φ = 0.082).

Dogs in the SRB group received toys from their holders significantly more frequently (χ^2^ (1, *N* = 851) = 11.529, *p* < 0.001) during separation than dogs without SRB (SRB: 41.18%, n = 85; non-SRB: 23.63%, n = 766). Food was also offered more often to SRB dogs (χ^2^ (1, *N* = 850) = 12.866, *p* < 0.001) (SRB: 42.35%, n = 85; non-SRB: 23.79%, n = 765). The effect size for both variables was weak (Toy: φ = 0.116; Food: φ = 0.123).

There were clear differences between the two groups regarding the greeting situation. Firstly, dogs that did not show SRB were greeted by their holders significantly more often (89.49%, n = 780) than SRB dogs (77.53%, n = 89) (χ^2^ (1, *N* = 869) = 9.904, *p* = 0.002, φ = 0.107). In addition, both groups also differed significantly regarding the type of greeting they received (χ^2^ (3, *N* = 869) = 12.648, *p* = 0.005, φ = 0.121). Dogs from the SRB group were more often not greeted at all (SRB: 22.47%; non-SRB: 10.51%) and less often verbally and physically greeted (SRB: 56.18%; non-SRB: 68.59%) (Figure 7C).

With an average of 8.15 min (SD = 7.81) of relaxation time after a separation for SRB dogs, SRB dogs significantly differed from the other group with a mean of 4.3 min (SD = 3.54). The difference was significant with a *p* value of <0.001 and the effect size of 0.936 (d) was strong.

The evaluation of the LAPS showed no significant differences between holders of SRB and non-SRB dogs (total attachment: tWelch (106.78) = −0.657, *p* = 0.981; general attachment: tWelch (106.12) = 1.306, *p* = 0.194; animal welfare and rights: tWelch (108.66) = −1.043, *p* = 0.300; people substituting: tWelch (105.63) = −0.892, *p* = 0.374).

**Figure 8:**
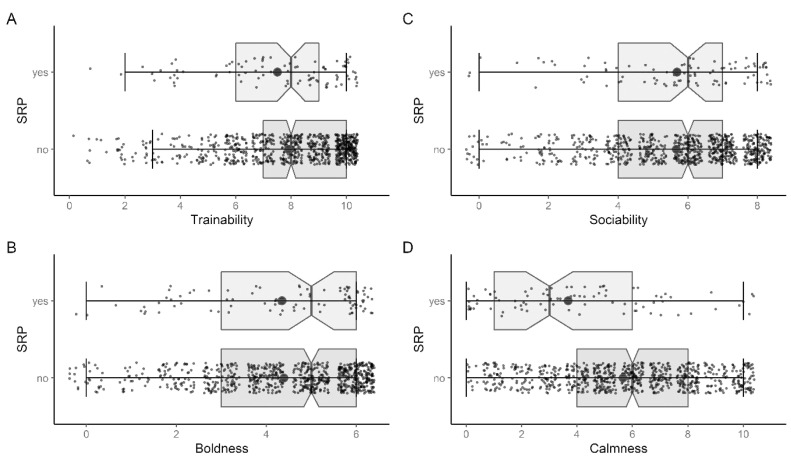
Group differences concerning (**A**) trainability (n = 869), (**B**) boldness (n = 869), (**C**) dog sociability (n = 869) and (**D**) calmness (n = 869).

The personality of the dogs only differed significantly between groups for calmness (tWelch (105.68) = 6.047, *p* < 0.001, d = 0.723) (Figure 8). With 5.66 points (SD = 2.72), non-SRB dogs were calmer than SRB dogs, with 3.67 (SD = 2.95) (Figure 8D). Cohen’s d did show a medium effect with 0.723. All other personality traits showed no noticeable differences (trainability: tWelch (106.43) = 1.525, *p* = 0.130; extraversion: tWelch (104.22) = 0.210, *p* = 0.834; dog sociability: tWelch (106.95) = −0.080, *p* = 0.937).

**Figure 9:**
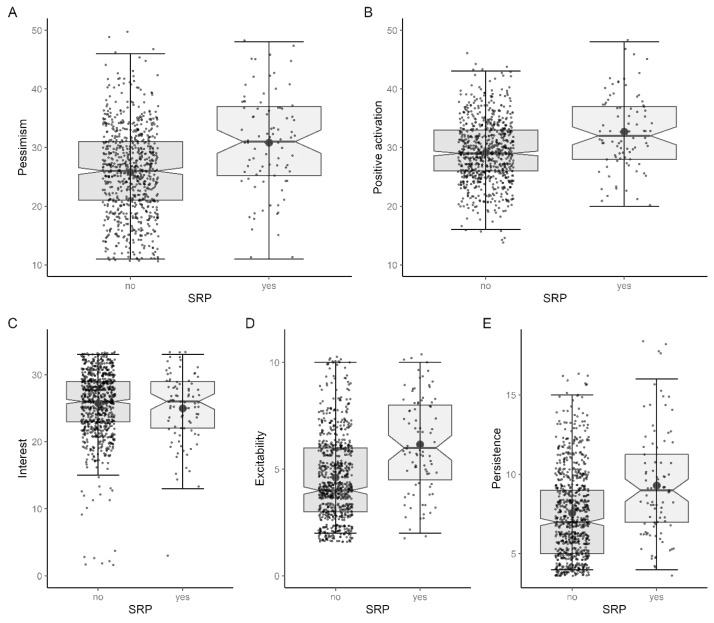
Group differences concerning (**A**) pessimism (n = 833), (**B**) positive activation (n = 840), (**C**) interest (n = 848), (**D**) excitability (n = 862) and (**E**) persistence (n = 855).

For emotional predisposition, SRB dogs scored higher than non-SRB dogs on all scales (Figure 9A,E). On the pessimism scale, non-SRB dogs scored 25.82 (SD = 7.37) on average, while SRB dogs scored 30.84 (SD = 8.33), representing a significant difference with a medium effect size (tWelch (100.91) = −5.355, *p* < 0.001, d = 0.672) (Figure 9A). The positive activation was also significantly (tWelch (100.66) = −5.052, *p* < 0.001, d = 0.654) higher in the SRB group than in the other dogs (M = 25.82, SD = 7.37) with 32.68 (SD = 6.25) (Figure 9B). On the three subscales of positive activation, both groups were very similar on the interest scale (tWelch (105.41) = −1.051, *p* = 0.296) (Figure 9C). On the scales of excitability and persistence, SRB dogs had higher scores and a significant difference with a medium effect size was evident (excitability: tWelch (101.18) = −6.244, *p* < 0.001, d = 0.788; persistence: tWelch (99.74) = −4.499, *p* < 0.001, d = 0.612) (Figure 9D,E).

## 4. Discussion

### 4.1. Test

Not unexpectedly, dogs spent most of the time in passive behaviors such as lying alert and resting during the test. Concerning behavior groups, attentive behavior was the most frequent and physical activity was rare. Stress-related behavior and vocalization was even rarer. Similar results were also found in other studies [2,6]. The researchers reasoned that the dogs might not have been motivated to show signals since no human was present, and the effort would outweigh the benefits. The counterargument was that dogs with separation anxiety show more physical activity and vocalization [2]. This thought will be continued here. If a dog is afraid of separation and therefore shows increased physical activity and vocalization during the separation, the underlying emotion, and thus physiology, is different. Dogs that are afraid to separate release stress hormones, which in turn have an activating effect. The level of suffering that dogs with such a problem have is not comparable in intensity. Escaping the perceived danger takes the highest priority and makes the willingness to send out signals, and thus the application of energy, more understandable. The motivation is therefore different in terms of orientation and intensity. Dogs that do not have separation anxiety are in a different state, hormonally and emotionally. It is therefore conceivable that the motivation to send out signals would also be lower, and the observed passive behavior would be understandable. Mazzini et al. [40] studied howls of captive wolves. They found that the relationship quality between the howler and the leaving individual was more predictive of howling occurrence than cortisol levels. It was then suggested that howling may be more controlled and used flexibly, influenced by the animal’s perception of its social world [40]. The missing connection between howling and cortisol may indicate that vocalization in dogs without separation problems is more likely to be influenced by cognitive factors and used in a targeted manner. However, it is also conceivable that the different types of vocalizations have different meanings and triggers. This connection will be discussed in more detail later.

Differences in behaviors between the different time intervals could only be detected in the pre-separation interval and in the first 5 min of separation. Only the behaviors of lying resting and physically active were significantly different. Lying resting was at its lowest after the shortest separation, but second highest after the longest separation. The highest value was reached after about 4 h of separation and not after 6 h. While physical activity was most evident after the longest period of separation, it was lowest after 4 h, rather than just 2 h. In the E1 interval, there was only a clear difference regarding walking, in which the highest level was found after the longest separation period. No significant effects could be found for all other behaviors and intervals. The differences during the pre-separation period and E1 were not related to the duration of the separation, since in these intervals the dog could not know or experience that the separation could last longer. The fact that no other differences were found for the intervals of separation was consistent with the literature, whereby it has been repeatedly stated that the activity of dogs without separation problems was low during separation or even decreasing, and not increasing with longer separation duration [2,6,10]. It was surprising that no differences were found for the time after the separation, since in the study by Rehn and Keeling [2] the dogs showed more intensive greeting behavior after a longer separation.

In addition, it was examined what influences there can be on behavior during the test situation. The attachment of the holder, as well as the personality and emotional predisposition of the dog, resulted in connections to some of the behaviors shown. Attentive behavior was more frequent in more extraverted, less calm dogs and dogs with more attached to their holders. Holders interacted more with their dogs if they were less positively activated and more social. Although vocalization was infrequent, dogs that were more positively activated were more likely to exhibit this behavior. More extraverted dogs exhibited less stressful behavior, and dogs whose holders were more attached to them showed more interactions with the holder. Even if there were no significant results after Holm–Bonferroni correction, the relationships are discussed here anyway, since the effect sizes were all strong, the sample size was very small and, therefore, significant results were difficult to achieve [41,42,43].

It was evaluated that the more the holder was attached to the dog, the more frequently the dog interacted. Konok et al. [16] found that dogs behave differently after separation towards their holder depending on the attachment style. Dogs with a secure attachment to the holder greeted the most intensively and thus interacted the most with the holder. It could also be assumed that holders who had achieved a high attachment score in the test were also more likely to engage in interactions with their dog, which could lead to the dog expecting interactions to be reciprocated. An important characteristic that defines the existence of attachment is that both partners seek proximity. This could be reflected in the relationship between attachment and interaction by the dog.

Dogs described by their holders as more extraverted and calmer were less likely to show attentive behavior. Apparently, the holders assessed their dogs realistically. Konok et al. [1] were able to show that more anxious dogs tended to be more active during the separation. Since extraversion and calmness indicate that a dog is less fearful and less stressed, it made sense that attentive behavior, i.e., activity, could be less common during the separation when these traits were more pronounced. The positive connection between attachment by the holder and attentive behavior during and after the dog’s separation could have something to do with the fact that securely attached holders communicated with their dog more often and the dog was therefore more attentive towards the holder or the leaving of the holder.

It makes sense that stress behaviors decrease the more extraverted a dog is, since the dog is less fearful during separation. The connection between the positive activation of the dog and the more frequent interactions by the holder may have had something to do with the fact that these dogs were generally more persistent and interested on the subscales, and the holders were therefore more inclined to interact with them. Conversely, when dogs were less social, holders interacted less frequently with the dogs. The fact that increased persistence can be associated with more frequent vocalization has already been mentioned and may be responsible for the strong effect size of positive activation and vocalization [44].

Using the cortisol difference values showed that the difference was positive after the separation but was not significant. Since it is difficult to achieve significant results with small sample sizes, as in the test we performed [43], it is worth pointing out that there were differences. The correlation between the length of separation and the difference in cortisol showed no significance and just a small effect. The results indicated that physiological stress did not increase significantly with duration, but there was a small effect. In the study by Rehn and Keeling [2], no physiological reaction to the duration of the separation could be found.

### 4.2. Online Survey

The proportion of dogs with SRB in this study was significantly lower than has been reported before. Since only dogs that showed one of the symptoms at least frequently were counted as SRB dogs, the proportion may have been higher because not all of the symptoms are always shown or they may not have been noticed by the holders. It is possible that the proportion is still lower than is generally known from the literature, as more committed dog owners probably took part in the study, as it can be assumed that they are more likely to be in dog groups on Facebook to deal with topics related to dogs.

The questioning regarding the symptoms included behaviors that, according to the literature, are typical for separation-related problem behavior. Not surprisingly, SRB dogs showed all symptoms more frequently. It was interesting that only some behaviors had at least a medium effect: restlessness, excitement, whining, howling, lip licking, barking and salivation.

The increased occurrence of restlessness and excitement was understandable. These behaviors probably represented a collective term for behaviors and left room for interpretation as to which behaviors can be meant by them. It can be assumed that holders also meant motor activities when interpreting the terms. Studies have shown that dogs with separation problems are more physically active than dogs without separation problems [2,9,10].

Barking, whining and howling were more common in SRB dogs. In this work, barking was shown most frequently, while whining and howling occurred somewhat less frequently, but still frequently. Similar results were found in a study by Lund and Jørgensen [10]. While this study did not distinguish which internal state, e.g., fear, caused the separation problem, Pongrácz et al. [11] investigated the occurrence of different types of vocalizations in dogs with separation anxiety. It was found that dogs who were afraid of separation whined more often than dogs without separation anxiety. Barking was independent of the grouping and was therefore not typical of separation anxiety [11]. The researchers hypothesized that different internal states might be behind the different types of vocalizations, suggesting that barking occurs in frustrated dogs and whining in fearful dogs [11]. Lund and Jørgensen [10] already had the idea that separation causes frustration in dogs because an important resource, the holder, becomes unreachable. This frustration leads to arousal in the dog, which causes increased barking and exploratory behavior. Depending on a dog’s previous experiences, this frustration and excitement can in turn lead to fear and thus fear-related behaviors such as whining [10]. Complementing these thoughts, the study by Lenkei et al. [44] is very interesting. It was examined to what extent fearfulness and demanding behavior affects vocalization. It was found that more fearful dogs whined earlier and more. Dogs that scored high on the demanding scale barked sooner and more frequently. Demanding behavior meant persistent behavior regarding food or play. The researchers assumed that demanding dogs have high expectations and when these are not met, for example, because the reward is not forthcoming or the holder is still not back, frustration erupts. A strong correlation between frustration and persistence was assumed. If the demanding dogs do not achieve their goal, they will keep trying to achieve it, using barking to do so [44]. The connection between separation and howling has already been mentioned before [40]. Separation problems can thus be divided into categories depending on the inner state and motivation of the dog. This subdivision was not made in this work, so the frequent occurrence of all three types of vocalizations was understandable. However, it has been shown that whining, barking and howling play a role in identifying separation problems.

Displacement activities such as lip licking were observed more frequently in SRB dogs. This result was not surprising as this behavior has also been implicated in the occurrence of separation problems in other studies [10]. Lip licking can be an indicator of stress; hence, it makes sense that this can play a role in the presence of separation issues.

The increased occurrence of salivation in SRB dogs was expected, as this had already been noticed in other studies [15,17,21]. As separation issues can have a variety of causes, excessive salivation may be more related to a panic or anxiety response.

Destruction has been cited and observed repeatedly in studies of separation problems in dogs [9,10,15,17,21]. On the one hand, destruction could take place out of frustration, but destruction in connection with escape behavior is also conceivable, e.g., by damaging doors. Since damage in the home is always noticed by the holder, this behavior was probably also realistically presented in terms of frequency. In the present work, destruction occurred significantly more frequently in SRB dogs, but only a small effect size could be found.

Urination and defecation were rare. This result was surprising, since both behaviors are assumed to be typical for separation problems [15,17,21]. It is possible that urination and defecation play a subordinate role in separation problems, as has been reported in some studies [9,10]. The importance of vocalization, and not defecation and urination, is repeatedly mentioned [9,10]. Aside from separation issues, defecation and urination can also result from holders not giving their dog enough opportunity to defecate. Since more committed dog holders probably took part in the questionnaire, it is assumed that this was rarely the case here.

The question of which factors can influence symptoms was also evaluated. It was analyzed in which characteristics the dogs which, according to the questionnaire, often or frequently showed symptoms during the separation, differed from the other dogs.

Mixed breeds were more often SRB dogs. In addition, they were more likely to come from national and international animal shelters, less often from breeders and more often had a stay in a shelter. In a study by McGreevy and Masters [19], no effect was found and purebred and mixed breed dogs did not differ in SRB status. The study was conducted in Australia, which may account for the inconsistency. However, there are some studies where the opposite was the case [14,45,46]. Pongracz et al. [11] reported that more mixed breeds whined, but purebred and mixed breeds barked sooner, thus suggesting that whining may be a better indicator of separation problems. As mixed breeds are much more likely to come from an animal shelter than purebred dogs, the effect of these variables on SRB status was understandable. In their study, Flannigan and Dodman [14] were also able to show that dogs that came from a shelter had more separation problems than dogs from a breeder. A connection to high pessimism values, which will play a role later, would be conceivable here. Dogs that come from shelters are mostly mixed breeds and could often reach high pessimism scores through their experiences. Another connection between mixed breeds and the personality trait calmness was found, which will also play a role later. According to Turcsán et al. [46], mixed breeds are less calm than purebred dogs.

Interestingly, on average, SRB dogs were separated from their holders less frequently and for shorter periods. The difference between the two groups in terms of duration and frequency of separation was not surprising. Presumably, dogs that were left alone more regularly were more used to it and the regularity increased predictability, which in turn provided reassurance. A study by Scanlon et al. [25] was able to show that dogs of homeless people who are rarely alone are more likely to have separation problems.

There were some differences between the two groups regarding the location during the separation. SRB dogs were less often indoors, more often had only one room and less often several rooms available and were more often kept in an inside kennel during the separation. This effect could have come about because, in addition to the separation, dogs are also confronted with changes that contribute to their discomfort. Most dogs that lived indoors were allowed to use all rooms when the human was at home with at most a few restrictions. If the separation from the holder is combined with a change in the dog’s environment, this could contribute to the dog experiencing the situation in a rather negative way. Locking up a dog in a kennel is relevant to animal welfare, since the dog’s movement is completely restricted over a longer period. It is legally prohibited in Germany, except for transport or vet treatment. Dogs that were outside during the separation may have fewer opportunities to withdraw and therefore feel more insecure. Additionally, they may have been exposed to external stimuli more often, such as noise, to which they react, and which make them less likely to settle down.

SRB dogs were more frequently with another pet during the separation, and toys and food were available to them more often. Another pet could make the situation even more difficult as the activity could be unsettling or at least activating for the dog. In a study by Stephan et al. [6] it was found that dogs that were with another animal during separation were more activated and vocalized more frequently. Some dog trainers recommend giving the dog toys or food when saying goodbye so that the dog associates the separation with something positive. These results revealed that holders who perform this ritual were more likely to have SRB dogs. Perhaps the toy or food acts as a key stimulus, letting the dogs know what is going to happen next, thus creating tension. Another thought is that dogs are distracted by the food or toy and not consciously aware of the holder leaving the home. Frustration settles in when perception returns to the environment and there are no more distractions.

It was not only the case that holders said goodbye to SRB dogs less often, but the greeting was also less frequent and less intense. In addition, SRB dogs received fewer verbal and physical greetings compared to non-SRB dogs. The greeting of the dogs was more intense when they belonged to the SRB group. In addition, the dogs needed more time to calm down after the holder’s return. The dog’s behavior during the leaving and greeting of the holder could play a role in the development of separation problems, since on the one hand it enables the holder to be predictable, which in turn can offer security for the dog, but in relation to the greeting it can also compensate for stress and thus cause a pleasant effect to arise, since oxytocin is released when they interact with each other. A study by Rehn et al. [7] showed that this effect is stronger with verbal and physical greetings than no greeting or only verbal greetings. Konok et al. [1] had conflicting results concerning the greeting intensity, since dogs with separation problems were not significantly more likely to show affection to their holders. Parthasarathy and Crowell-Davis [47] also found that post-separation proximity seeking did not differ between dogs with and without separation problems. However, there is also evidence to the contrary: Appleby and Plujimakers [34] and Flannigan and Dodman [14] found that dogs with separation problems greeted very intensely. Although the intensity of the greeting of the dog and the holder in the present work differed between SRB and non-SRB dogs, it is pointed out that the correlations were all small and thus may not have a strong effect on the assessment of separation problems. Possible interactions should of course be kept in mind. SRB dogs took about twice as long to relax. Konok et al. [1] had similar results, noting that dogs with separation problems were significantly more active (running) after separation than dogs without separation problems. The researchers assumed that this could indicate an insecure, ambivalent attachment to the holder, since despite the activity, there was no frequent seeking for proximity. A further thought is that dogs with separation problems were also more active during the separation and had more stress, and that this was reflected in physical activity for a period of time after the separation. While greeting characteristics achieved only small effect sizes, a strong association was found for time to relaxation. Thus, this variable may be more meaningful in assessing separation problems.

The personality of the dogs was also related to the SRB status. While trainability, dog sociability and extraversion showed no significant effects, dogs with SRB scored significantly lower on the calmness scale with a medium and almost strong effect. The calmness scale asked for emotionally balanced, calm, cool-headed, not anxious and not stressed behavior [38]. It was not surprising, then, that dogs that scored high on this scale also tended to do well during a separation. Furthermore, in relation to Tiira et al. [18], the result made sense because dogs with a high calmness score were probably less fearful and the general fearfulness was observed in connection with separation problems.

SRB dogs were more often more pessimistic and positively activated. Even if it seemed contradictory at first glance, the higher levels of pessimism and positive activation in SRB dogs were understandable. Looking at each subscale of positive activation, interest did not play a role, but excitability and persistence were more pronounced in SRB dogs. Lenkei et al. [44] found that the persistence and demanding behavior of the dogs was related to the frequency of barking during separation. Since barking was one of the vocalization types used to determine SRB status, this result makes sense. Presumably persistent dogs try longer to achieve their goal, in the situation of separation, to establish proximity to the holder. Mendl et al. [12] examined the difference in affective states in dogs with separation problems. It was found that pessimistic dogs were more likely to suffer from separation problems. Pessimism could lead to stimuli and situations, such as separation from the holder, being judged more negatively and perceived more often as potentially dangerous.

## 5. Conclusions

In the course of this work, many insights into the separation behavior of domestic dogs could be gained. Dogs without separation problems have been found to be mostly inactive during separation. If there was activity, then above all, attentive behavior played a role. To assess behavior during the separation, it makes sense to include personality traits and emotional disposition. Here, particular attention should be paid to extraversion, calmness and persistence be able to better assess emotional states during the separation. The duration of separation had no effect on the behavior in dogs without separation problems but did show a slight effect on cortisol levels. It therefore makes sense to include the cortisol measurement in the assessment of the separation behavior to gain a more detailed impression. Possible influences on the cortisol concentration should be controlled and standardized as far as possible.

Separation behavior in SRB dogs differed significantly from dogs without separation problems. The differences became clear above all through the increased physical activity and vocalization. The type of vocalization also played a role and could give an indication of various causes and influences. Many of the possible influencing factors on the SRB status were significant, but most had negligible or small effects, and interactions are possibly influential. The time it takes to relax after a separation has been found to be a strong indicator of the presence of separation problems. Again, it would be useful to include personality traits and emotional disposition. Unlike dogs without separation problems, pessimism was important here. Apart from that, frequency of separation, as the second-strongest effect after time to relaxation, was shown to be a factor that may influence the occurrence of separation problems. It is conceivable that the regularity of a separation is important to avoid separation problems. The importance of greeting when separation problems are present became clear. A greeting that takes place verbally and physically seems to ensure that separation problems are less likely to occur. It also became clear that it is necessary to take a multifactorial approach to better understand the separation behavior of dogs.

## Figures and Tables

**Table 1:** Behavioral catalogue adapted from Rehn and Keeling [2].

Behavior	Definition
Lying alert ^b,g^	Dog lying down, head not in contact with floor
Lying resting ^g^	Dog lying down, head in contact with floor
Sitting ^g^	Dog sitting with front legs extended and hind legs curved
Standing ^g^	Dog standing up on all four paws
Walking ^g^	Dog walking around/moving
Running ^a,g^	Dog running around, trotting, or galloping
Exploring ^b^	Motor activity directed towards any physical aspect of environment
Attention towards something ^b^	Dog has ears and eyes pointed in certain direction (starting > 2 s), e.g., towards door, window, etc.
Play ^a^	Any vigorous or galloping gaited behavior directed towards a toy
Grooming ^a^	Dog cleaning its body surface
Chewing ^a^	Dog chewing an object or eating/drinking
Panting ^a,f^	Increased frequency of inhalation and exhalation with mouth open
Tail wagging ^a,f^	Repetitive wagging movement of tail
Barking ^a,e,f^	Dog barking
Growling ^a^	Dog growling
Whining ^a,e,f^	Dog whining
Howling ^a,e,f^	Dog howling
Yawning ^b,f^	Dog opens its mouth widely and inhales
Lip licking ^b,f^	Dog snout licking, tongue visible
Body stretching ^f^	Dog extending/stretching part of or whole body
Body shaking ^f^	Dog shakes any part of or whole body from side to side
Following holder ^a,c^	Walking behind holder within distance
Physical contact ^b,c^	Dog leans, jumps up on, and/or nudges/licks holder
Attention towards holder ^b,c^	Dog focused on holder by gazing/staring at holder (>2 s)
Inviting play ^a,c^	Dog initiates play with holder (<0.5 m)
Holder physical contact ^d^	Holder pets/strokes/scratches dog
Holder verbal contact ^d^	Holder talks to dog
Holder invites play ^d^	Holder offers play, with or without toy
Holder seeks contact ^d^	Holder bends down towards dog or sits/lays down on floor

^a^ Physically active. ^b^ Attentive behavior. ^c^ Interaction by dog. ^d^ Interaction by holder. ^e^ Vocalizing. ^f^ Stress behavior. ^g^ Restlessness.

## Data Availability

The data presented in this study are available on request from the corresponding author.

## Supplementary Materials

The following supporting information can be downloaded at: https://www.mdpi.com/article/10.3390/ani13233735/s1, Figure S1: Instructions video recording; Figure S2: Instructions salivary sampling; Figure S3: Protocol; Table S1: Risk factors for separation-related behavior; Table S2: Separation questionnaire; Table S3: Descriptive statistics of behaviors in total of time; Table S4: Descriptive statistics of behavior groups in total of time; Table S5: Comparison of behaviors of different separation durations; Table S6: Effect sizes and *p* values on SRB status.
