# Supplementary material for: Factors Influencing Isolation Behavior of Dogs: A Holder-Based Questionnaire and Behavioral and Saliva Cortisol Responses during Separation
Source: Animals (Basel). 2023 Dec 2;13(23):3735. doi: 10.3390/ani13233735 (PMC10705200; doi:10.3390/ani13233735)
Supplement: Supplementary file 1 [file animals-13-03735-s001.zip › animals-2661349-Supplementary Material.pdf]

Figure S1. Instructions video recording.

Leitfaden zur Videoaufnahme

erstellt von: Jennifer Silbermann

Technische Voraussetzungen

Da Ihr Hund für mehrere Stunden aufgenommen werden soll, ist es wichtig, dass das Medium über ausreichend Speicherplatz verfügt. Je nach Gerät und Format können Dateien von bis zu 12 GB entstehen. Am einfachsten ist es ein Notebook mit integrierter Kamera zu nutzen. Tablet oder Handy können ebenso verwendet werden, vorausgesetzt es ist genügend Speicherplatz und stetige Stromzufuhr vorhanden.

Probelauf

Damit am Aufnahmetag nichts schief geht, empfehle ich dringend die Technik vorher einmal Probe laufen zu lassen. Bestenfalls nehmen Sie dazu über 6 Stunden auf und kontrollieren danach, ob alles geklappt hat. Manche Geräte besitzen leider eine maximale Aufnahmedauer, die unter 6 Stunden liegt. Auch deshalb ist ein vorheriger Test sehr sinnvoll.

Zur Aufnahme können Sie die bereits vorhandene Kamera-Software Ihres Gerätes nutzen, aber auch die Verwendung von kostenloser Software, wie VLC-Player, sind möglich.

Falls Sie vor oder nach der Aufnahme nach einem Speicherformat gefragt werden, wählen Sie gerne MP4 aus. Falls nicht, ist das auch in Ordnung, dann einfach nur speichern.

Ganz wichtig: Gerät dauerhaft am Strom lassen. Auch der Stromsparmodus sollte ausgeschaltet sein.

Platzierung der Kamera

Falls sich Ihr Hund nur in einem Raum frei bewegen kann, platzieren Sie die Kamera bitte so, dass ein möglichst großer Bereich des Raumes sichtbar ist.

Darf sich Ihr Hund, während der Trennung, gänzlich frei in allen Räumen bewegen, platzieren Sie die Kamera bitte so, dass der Ort, an dem sich Ihr Hund Ihrer Vermutung nach meistens aufhält, aufgenommen wird.

Wichtig ist, dass auch der Ton mit aufgezeichnet wird, damit Lautäußerungen wie Bellen oder Heulen, in die Auswertung einfließen können.

Es ist außerdem bedeutend, dass die Begrüßung zwischen Ihnen und Ihrem Hund nach Ihrer Rückkehr gut sichtbar ist. Sollte die Kamera den Eingangsbereich nicht mit abbilden, bitte ich darum den Hund, soweit / so schnell wie möglich, im gefilmten Bereich zu begrüßen.

Falls Ihnen sogar zwei Kameras zur Verfügung stehen, können Sie gern eine Kamera im Lieblingsraum und eine im Eingangsbereich platzieren. Dann müssen Sie sich über Ihren Standort während der Begrüßung keine Gedanken machen.

Ablauf

Wir sind daran interessiert herauszufinden, ob sich das Verhalten Ihres Hundes ändert, wenn die Dauer der Trennung steigt.

Ihr Hund wird deshalb über drei Zeitintervalle aufgenommen: 2, 4 und 6 Stunden.

Inklusive der 10-minütigen Aufnahme vor Ihrem Verlassen und nach Ihrer Rückkehr, ergeben sich Aufnahmezeiten von mindestens 2:20, 4:20 und 6:20.

Nötige Angaben halten Sie bitte im Protokoll fest.

Starten der Aufnahme

Die Aufnahme startet mindestens 10 Minuten vor Ihrem Verlassen, so dass wir analysieren können, wie sich Ihr Hund in dieser Zeit verhält. Führen Sie während dieser Phase Ihre normalen Routinen aus und verhalten Sie sich wie sonst auch.

Danach bleibt Ihr Hund für 2, 4 oder 6 Stunden allein. Mit welcher Dauer Sie beginnen, bleibt Ihnen überlassen.

Beendigung der Aufnahme

Die Aufnahme stoppt mindestens 10 Minuten nach Ihrer Rückkehr. Uns interessiert besonders wie sich Ihr Hund während der Begrüßung und kurze Zeit danach verhält. Machen Sie auch hier einfach alles wie immer. Falls die Kamera nicht so platziert ist, dass auch der Eingangsbereich abgebildet wird, ist es wichtig darauf zu achten sich zügig im gefilmten Bereich aufzuhalten, so dass das Begrüßungsverhalten so umfassend wie möglich erfasst werden kann.

Benennung Datei

Ihre Initialen + Ihres Hundes + Intervalldauer

Beispiel: Max Mustermann hat seinen Hund Peppy über 6:20 Stunden allein Zuhause aufgenommen: MMP6

Ggf. Komprimieren

Falls Ihre Internetverbindung keine hohe Übertragungsrate hat und Sie die Zeit des Hochladens gern verkürzen möchten, ist es möglich das Video im Vorfeld zu verkleinern. Dazu gibt es verschiedene kostenfreie Programme. Mehr Infos dazu finden Sie in der Datei „Komprimierung_Videos".

Hochladen der Videos

Die Videos werden auf einem sicheren Nextcloud-Server gesammelt und abgerufen. Nur der Admin hat darauf Zugriff. Nach Beendigung der Projektarbeit werden alle Videos gelöscht.

Unter folgendem Link können Sie Ihre Aufnahmen hochladen:

https://rv2950.1blu.de/index.php/s/rgjiYXtwJBF3WnT

Die Upload-Dauer wird einige Zeit (mehrere Stunden) in Anspruch nehmen. Es empfiehlt sich daher dies zu machen, wenn das Gerät für längere Zeit angeschaltet bleiben kann.

Alternativ ist es möglich die Daten auf einem Stick im Paket mit den Speichelproben zu schicken. Ihr Stick wird Ihnen dann zeitnahe per Brief zurückgesendet.

Zeitraum

Da das Projekt an Fristen gebunden ist, können nur Aufnahmen berücksichtigt und ausgewertet werden, die bis einschließlich 22.04.2022 hochgeladen / zugesendet wurden.

Bei jeglichen Fragen oder Problemen wenden Sie sich gerne an **projectconnect@outlook.de**.

Figure S2. Instructions salivary sampling.

Leitfaden zur Speichelproben-Entnahme

erstellt von: Jennifer Silbermann

Wir sind daran interessiert herauszufinden, ob sich die Konzentration des Stresshormons Cortisol Ihres Hundes ändert, wenn die Dauer der Trennung steigt. Dazu werden Speichelproben Ihres Hundes genommen.

---

vorheriges Training

Um ein gutes Ergebnis der Speichelprobe zu erreichen, empfiehlt es sich **mindestens 7 Tage** vor der ersten Testung, **3 bis 5 Mal täglich** mit dem Hund zu trainieren.

Während der Durchführung macht es sich am besten, wenn ihr Hund sitzt und Sie sitzen/hocken. Anfangs wird die Watte nur gezeigt, noch nicht ins Maul gelegt, Sie loben Ihren Hund verbal und der Hund bekommt eine Belohnung, sobald dieser sich der Watterolle in irgendeiner Form nähert oder diese berührt. Nach einigen Wiederholungen kann es nun einen Schritt weiter gehen. Nehmen Sie die Watterolle und legen Sie diese in die Backentasche (siehe Abbildung 6 unter „Vorgehen“). Falls Sie befürchten, dass Ihr Hund die Watterolle verschlucken könnte, ist es auch möglich, dass Sie diese am Ende festhalten, während sich das andere Ende in der Backentasche befindet. Dort lassen Sie sie nur für einen kurzem Moment, entnehmen diese, loben Ihren Hund verbal (falls Sie schon ein Markersignal konditioniert haben, können Sie gerne auch dieses nutzen) und belohnen den Hund gleich danach mit Futter.

Bewahren Sie das Leckerli während der Testung bitte nicht einsehbar für Ihren Hund auf, damit dieser durch den Anblick nicht zu unruhig wird.

Die Zeit bis die Watterolle entnommen wird, wird in kleinen Schritten von **etwa 5-10 Sekunden** verlängert, vorausgesetzt der Hund zeigt keine Unruhe, während die Watte in der Backentasche ist.

Sobald Ihr Hund die Watterolle für **mindestens 30 Sekunden** in der Backentasche toleriert, nutzen Sie die **letzten 5 Sekunden** bitte dazu, dass Sie die Watterolle unter der Zunge des Hundes entlangführen (dort sammelt sich besonders viel Speichel).

Der Hund sollte letztendlich kennen, dass die Watte mindestens **1 bis bestmöglich 2 Minuten** im Maul bleibt. Falls die Rolle einmal ausgespuckt wird oder herausfällt, kann diese einfach wieder nachgelegt werden.

Bitte hören Sie mit dem Training bestenfalls auf bevor Ihr Hund unruhig wird.

Ihr Hund sollte die Watterolle nach dem Training mit Futter in Verbindung bringen und damit positiv verknüpft haben. Damit es dabei bleibt, bitten wir darum, die **Testtage der drei verschiedenen Intervalle nicht hintereinander** zu legen. Der Hund könnte durch den vorangegangenen Tag lernen, dass Watterolle in der Maultasche bedeutet, dass der Mensch danach geht. Daher könnte schon der Anblick der Watterolle das Stresshormon beeinflussen.

Lassen Sie deswegen bitte zwischen den „echten" Testtagen **mindestens zwei Tage ohne Testung stehen und konditionieren** Sie in dieser Zeit noch einmal nach (1-2 Minuten Watterolle + Belohnung UND Sie bleiben danach bei Ihrem Hund und haben eine entspannte Zeit).

Ablauf

Anders als bei den Videoaufnahmen, werden die Speichelproben **nur für zwei Intervalle** gemessen: **2 und 6 Stunden**.

Dazu ist es nötig Ihrem Hund am Morgen der Testung, vor und nach dem Alleinbleiben Speichelproben zu entnehmen.

Der **Morgenwert** sollte möglichst zwischen **7 bis 10 Uhr** genommen werden. Ihr Hund sollte vor der Speichelprobe bestenfalls noch nichts gemacht haben (Trinken, Fressen, Bewegung).

Vor dem Alleinbleiben wird eine weitere Probe genommen. Nehmen Sie diese bitte bevor Sie beginnen sich zum Weggehen fertig zu machen.

Nachdem Ihr Hund 2 oder 6 Stunden allein Zuhause war, wird die letzte Probe genommen. Begrüßen Sie Ihren Hund gerne so wie sonst auch und beginnen danach mit der Speichelprobenentnahme.

Ihr Hund kann durch die Speichelproben-Entnahme hormonell beeinflusst werden. Daher ist es wichtig, dass die Entnahme (Schritt 6 s.u.) **nicht länger als 4 Minute**n dauert, um mögliche Verfälschungen der Ergebnisse zu vermeiden. Falls es doch einmal länger dauern sollte, vermerken Sie dies bitte auf dem beigefügten Protokoll.

Wichtige Hinweise:

- Nehmen Sie die Probe **am Morgen** bevor Ihr Hund gefressen, getrunken oder sich vermehrt bewegt hat

- Innerhalb von 30 Minuten vor der Messung **vor dem Alleinbleiben**, sollte Ihr Hund **nichts trinken und fressen**
- Innerhalb von 60 Minuten vor der Messung **vor dem Alleinbleiben**, sollte Ihr Hund sich **nicht intensiv bewegen**
- Watterolle für **mind. 1 bis bestmöglich 2 Minuten** in Backentasche des Hundes
- Schritt 6 sollte **nicht länger als 4 Minuten** dauern, sonst können Ergebnisse beeinflusst werden
- Vergessen Sie nicht Ihren Hund nach Speichelentnahme zu **belohnen**
- Füllen Sie nach jeder Speichelproben-Entnahme das **Protokoll** aus
- Die im Protokoll erfasste **Entnahmedauer** meint die Dauer für Schritt 6
- Sollte etwas anders laufen als gedacht oder es **besondere Vorkommnisse** vor der Messung geben (bsp. Streit unter Artgenossen, läufige Hündin beim letzten Gassigang, Futter kurz vor Speichelprobe, usw.), teilen Sie uns dies bitte im **Bemerkungsfeld** des Protokolls mit
- Legen Sie die Proben schnellstmöglich ins **Tiefkühlfach**

Und so geht's:

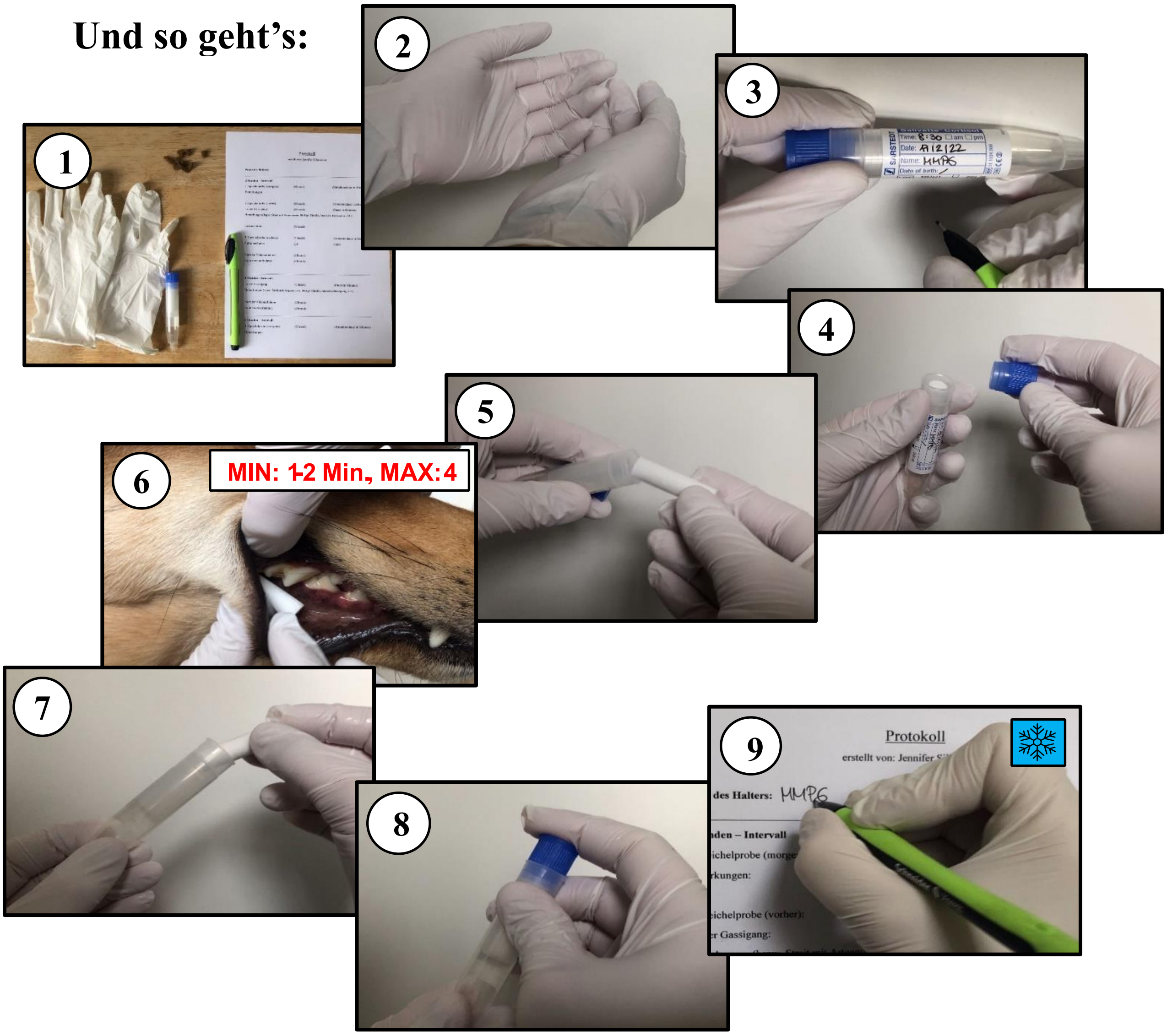

Beschriftung der Proben

Beschriften Sie die Röhrchen bitte mit Ihrer ID:

Ihre Initialen + Ihres Hundes + Intervalldauer

Beispiel: Max Mustermann hat seinen Hund Peppy über 6 Stunden allein Zuhause gelassen und nimmt nun eine Speichelprobe: MMP6

Versenden der Proben

Da für zwei Intervalle und je Intervall drei Proben genommen werden, sollten am Ende sechs Speichelproben vorhanden sein.

Das Versandmaterial und Materialien zur Probenentnahme wird Ihnen postalisch zugestellt. Darin enthalten sind sieben Probenröhrchen inkl. Watterolle (1x Trainingsprobenröhrchen, 6x Versuchsprobenröhrchen), Einweghandschuhe, alle nötigen Handzettel und Porto für den Rückversand. Bitte nutzen Sie für den Rückversand dieselbe Kartonage und senden alle 6 Proben gesammelt an Jennifer Silbermann, Steigerstr. 33, 99096 Erfurt als Maxibrief zurück. Die Proben sollten erst aus dem Tiefkühler genommen werden, wenn Sie die Proben versenden möchten, damit diese nur wenige Tage ungekühlt unterwegs sind. Sind die Proben mehr als 4 Tage ungekühlt, kann das Ergebnis verfälscht werden. Um das zu vermeiden, wird dringend empfohlen nicht vor dem Wochenende, sondern Anfang der Woche zu versenden.

Zeitraum

Da das Projekt an Fristen gebunden ist, können nur Proben berücksichtigt und ausgewertet werden, die bis einschließlich 22.04.2022 eingesendet wurden.

Bei jeglichen Fragen oder Problemen wenden Sie sich gerne an **projectconnect@outlook.de**.

Figure S3. Protocol.

<u>Protokoll</u>

erstellt von: Jennifer Silbermann

Name des Halters:

Medikamente:

Erkrankungen:

---

2 Stunden – Intervall

1. Speichelprobe (morgens): (Uhrzeit) (Entnahmedauer in Minuten)
   Bemerkungen:

2. Speichelprobe (vorher): (Uhrzeit) (Entnahmedauer in Minuten) letzter
   Gassigang: (Uhrzeit) (Dauer in Minuten)

Bemerkungen (bspw. Streit mit Artgenossen, läufige Hündin, intensive Bewegung, o.ä.):

letztes Futter: (Uhrzeit)

Start der Videoaufnahme: (Uhrzeit)

3. Speichelprobe (nachher): (Uhrzeit) (Entnahmedauer in Minuten)

Futter verfügbar während Trennung: (ja) (nein)

Ende Videoaufnahme: (Uhrzeit)

---

4 Stunden – Intervall

letzter Gassigang: (Uhrzeit) (Dauer in Minuten)

Bemerkungen (bspw. Streit mit Artgenossen, läufige Hündin, intensive Bewegung, o.ä.):

Start der Videoaufnahme: (Uhrzeit)

Ende Videoaufnahme: (Uhrzeit)

---

6 Stunden – Intervall

1. Speichelprobe (morgens): (Uhrzeit) (Entnahmedauer in Minuten)

Bemerkungen:

2. Speichelprobe (vorher): (Uhrzeit) (Entnahmedauer in Minuten) letzter

Gassigang: (Uhrzeit) (Dauer in Minuten)

Bemerkungen (bspw. Streit mit Artgenossen, läufige Hündin, intensive Bewegung, o.ä.):

letztes Futter: (Uhrzeit)

Start der Videoaufnahme: (Uhrzeit)

3. Speichelprobe (nachher): (Uhrzeit) (Entnahmedauer in Minuten)

Futter verfügbar während Trennung: (ja) (nein)

Ende Videoaufnahme: (Uhrzeit)

Table S1. Risk factors for separation-related behavior.

Author	Year	Factor
Konok et al.	2019	owners with few worries during separation
Konok et al.	2019	insecure dogs
Flannigan & Dodman	2001	single owners
Flannigan & Dodman	2001	intact dogs
McGreevy & Masters	2008	
Storengen et al.	2014	castrated dogs
Bradshaw et al.	2002	male dogs
McGreevy & Masters	2008	
McGreevy & Masters	2008	dogs from pet shops
McGreevy & Masters	2008	number of adult females in household
Mendl et al.	2010	pessimism in dog
Konok et al.	2011	intensity of greeting behavior of dog
Pongrácz et al.	2017	breed
Tiira et al.	2016	comorbidity phobias
Overall et al.	2011	
Konok et al.	2011	no calming down after owner's return

McCrave	1991	time in shelter
Scanlon et al.	2022	frequency of separation
Harvey et al.	2022	reduced separation duration
Palestrini et al.	2010	time of adoption
Palestrini et al.	2010	early separation from mother

Table S2. Separation questionnaire.

**Frage**	**Antwortmöglichkeiten**
Welches Geschlecht hat Ihr Hund? Ist er kastriert/sterilisiert?<br>(Mehrfachantwort möglich)	weiblich, männlich, kastriert, sterilisiert, Hormonchip (nicht länger als 12 Monate)
Wie alt ist Ihr Hund?	unter 4 Monate, 4 Monate bis 1 Jahr, 2 bis 5 Jahre, 6 bis 9 Jahre, 10 bis 13 Jahre, über 13 Jahre
Welcher Rasse gehört Ihr Hund an? Oder ist es ein Mischling aus …?<br>(Falls Rasse(n) nicht bekannt, bitte "Mischling" angeben.)	frei
Bitte geben Sie die Größe Ihres Hundes in cm (Schulterhöhe) an:<br>(Bitte nur Zahl angeben, z. B. 30)	frei

Frage	Antwortmöglichkeiten
Bitte geben Sie das ungefähre Gewicht des Hundes in Kilogramm an: (Bitte nur Zahl angeben, z. B. 25)	frei
Woher haben Sie Ihren Hund?	Züchter, Tierschutz - Inland/Tierheim, Tierschutz - Ausland, Bekannte/Verwandte, Sonstiges:
Falls Ihr Hund aus dem Tierheim/Tierschutz stammt, wissen Sie wie lange dieser dort war?	bis zu 1 Monat, bis zu 3 Monate, bis zu 6 Monate, bis zu 1 Jahr, mehr als 1 Jahr, weiß ich nicht
Wie lange ist Ihr Hund schon bei Ihnen?	unter 1 Monat, 1 bis 3 Monate, 4 bis 6 Monate, 7 bis 12 Monate, über 1 bis 3 Jahre, 4 bis 5 Jahre, mehr als 5 Jahre
Wie viele Halter gab es vor Ihnen?	ich bin Ersthalter, 1 Halter, 2 Halter, 3 bis 4 Halter, mehr als 4 Halter, weiß ich nicht
Wie alt war Ihr Hund als er zu Ihnen kam?	unter 1 Monat, 1 bis 2 Monate, über 2 bis 3 Monate, über 3 bis 5 Monate, 6 Monate bis 1 Jahr, 2 bis 5 Jahre, 6 bis 10 Jahre, 11 bis 15 Jahre, älter als 15 Jahre, weiß ich nicht
Welchem Geschlecht ordnen Sie sich zu?	männlich, weiblich, divers
Wie alt sind Sie?	18-25, 26-30, 31-40, 41-50, 51-60, älter als 61
Wie viele männliche Erwachsene leben in Ihrem Haushalt?	frei
Wie viele weibliche Erwachsene leben in Ihrem Haushalt?	frei
Wie viele Kinder (unter 14 Jahren) leben in Ihrem Haushalt?	frei
In welcher Art Unterkunft leben Sie? (Mehrfachantwort möglich)	Haus, Wohnung, mit Garten, Sonstiges:
Gab es in den letzten 12 Monaten in Ihrem Leben und in dem Leben des Hundes wichtige Veränderungen? (Mehrfachantwort möglich)	nein, Umzug, Scheidung/Trennung, Home Office, veränderte Arbeitszeiten, weniger Gassi als sonst, neuer Hund im Haushalt, Verlust eines tierischen Mitbewohners (Hund oder Katze), Geburt eines Kindes, Vorbesitzer verstorben, Sonstiges:
Lebt Ihr Hund mit einem oder mehreren Hund(en) zusammen?	ja, nein
Lebt Ihr Hund mit anderen Tierarten zusammen? Wenn ja, welche?	nein, Katze, Vogel, Nagetier, Sonstiges:

Frage	Antwortmöglichkeiten
Lassen Sie Ihren Hund manchmal alleine Zuhause?	ja, nein
Wie viele Tage in der Woche bleibt Ihr Hund alleine Zuhause?	1 bis 7 Tage
Wie lange lassen Sie Ihren Hund ohne Unterbrechung alleine Zuhause?	wenige Minuten, bis 1,5 Stunden, bis 2,5 Stunden, bis 3,5 Stunden, bis 4,5 Stunden, bis 5,5 Stunden, bis 6,5 Stunden, mindestens 8 Stunden
Wo befindet sich Ihr Hund während dieser Zeit?<br>(Mehrfachantwort möglich)	in einer geschlossenen Hundebox, Zugang zur/zum gesamten Wohnung/Haus, ausschließlich in einem Raum, mit Zugang nach draußen, ausschließlich draußen, Zwinger, Sonstiges:
Wie lange ist die Dauer zwischen dem letzten Gassigang und dem Alleinelassen des Hundes?	bis zu 5 Minuten, bis zu 30 Minuten, bis zu 1 Stunde, bis zu 3 Stunden, bis zu 4 Stunden, bis zu 5 Stunden, bis zu 6 Stunden, bis zu 7 Stunden, mehr als 7 Stunden
Wie lange dauert der letzte Gassigang vor dem Alleinelassen Zuhause (in Minuten)?<br>(Bitte keine Zeitspannen, sondern einen Durchschnittswert angeben)	frei
Hinterlassen Sie Ihrem Hund eine Beschäftigung für die erste Zeit des Alleinebleibens? Wenn ja, welche?<br>(Mehrfachantwort möglich)	nein, Futter, Spielzeug, Sonstiges:
Hat Ihr Hund einen anderen Hund, eine Katze oder ein anderes Haustier um sich, während er alleine Zuhause ist?<br>(Mehrfachantwort möglich)	nein, Hund, Katze, Sonstiges:
Für welche Dauer würden Sie Ihren Hund maximal alleine lassen?	wenige Minuten, bis zu 30 Minuten, bis zu 1 Stunde, bis zu 3 Stunden, bis zu 5 Stunden, bis zu 7 Stunden, bis zu 9 Stunden, mehr als 9 Stunden
Wie fühlen Sie sich, während Sie Ihren Hund alleine Zuhause lassen?<br>(1 = sehr ruhig, 2 = eher ruhig, 3 = eher unruhig, 4 = sehr unruhig)	1 bis 4
Wie glauben Sie fühlt sich der Hund, wenn Sie ihn alleine Zuhause lassen?<br>(1 = sehr ruhig, 2 = eher ruhig, 3 = eher unruhig, 4 = sehr unruhig)	1 bis 4

Frage	Antwortmöglichkeiten
Verabschieden Sie sich von Ihrem Hund bevor Sie Ihr Zuhause verlassen? Wenn ja, in welcher Form?	nein, mit Worten, körperlich, mit Worten und körperlich
Wie würden Sie Ihre Verabschiedung gegenüber Ihrem Hund einschätzen, bevor Sie Ihr Zuhause verlassen?<br>(1 = ich verabschiede mich nicht, 2 = sehr ruhig, 3 = eher ruhig, 4 = eher intensiv, 5 = sehr intensiv)	1 bis 5
Zeigt Ihr Hund während Ihrer Abwesenheit oder während Sie sich zum Verlassen fertig machen eine oder mehrere der folgenden Verhaltensweisen?	Weinen (nie, selten, manchmal, häufig, immer, weiß ich nicht),<br>Bellen (nie, selten, manchmal, häufig, immer, weiß ich nicht),<br>Heulen (nie, selten, manchmal, häufig, immer, weiß ich nicht),<br>Knurren (nie, selten, manchmal, häufig, immer, weiß ich nicht),<br>starkes Speicheln (nie, selten, manchmal, häufig, immer, weiß ich nicht),<br>Urinieren (nie, selten, manchmal, häufig, immer, weiß ich nicht),<br>Kot absetzen (nie, selten, manchmal, häufig, immer, weiß ich nicht),<br>Zerstörung Gegenstände (nie, selten, manchmal, häufig, immer, weiß ich nicht),<br>Zittern (nie, selten, manchmal, häufig, immer, weiß ich nicht),<br>Unruhe (nie, selten, manchmal, häufig, immer, weiß ich nicht),<br>Aufregung (nie, selten, manchmal, häufig, immer, weiß ich nicht),<br>Lippenlecken (nie, selten, manchmal, häufig, immer, weiß ich nicht),<br>Gähnen (nie, selten, manchmal, häufig, immer, weiß ich nicht),<br>Fluchtverhalten (nie, selten, manchmal, häufig, immer, weiß ich nicht)
Begrüßen Sie Ihren Hund, wenn Sie nach Hause kommen? Wenn ja, wie?<br>Wie intensiv würden Sie Ihr Begrüßungsverhalten gegenüber Ihrem Hund bei Ihrer Rückkehr einschätzen?<br>(1 = ich begrüße nicht, 2 = sehr ruhig, 3 = eher ruhig, 4 = eher intensiv, 5 = sehr intensiv)	nein, mit Worten, körperlich, mit Worten und körperlich<br>1 bis 5
Wie intensiv würden Sie das Begrüßungsverhalten Ihres Hundes einordnen, wenn Sie nach kurzer Zeit	1 bis 5

Frage	Antwortmöglichkeiten
(wenige Minuten) zurückkehren und Ihr Hund alleine Zuhause war?<br>(1 = begrüßt nicht, 2 = sehr ruhig, 3 = eher ruhig, 4 = eher intensiv, 5 = sehr intensiv)	
Wie intensiv würden Sie das Begrüßungsverhalten Ihres Hundes einordnen, wenn Sie nach längerer Zeit (mehrere Stunden) zurückkehren und Ihr Hund alleine Zuhause war?<br>(1 = begrüßt nicht, 2 = sehr ruhig, 3 = eher ruhig, 4 = eher intensiv, 5 = sehr intensiv)	1 bis 5
Wie schnell ist Ihr Hund schätzungsweise wieder in einem entspannten Zustand nachdem Sie zurück sind (in Minuten)?<br>(Bitte keine Zeitspannen, sondern einen Durchschnittswert angeben)	frei
In welchem Alter wurde Ihr Hund von seiner Mutter getrennt?	keine Trennung, sofort, innerhalb der ersten 4 Wochen, innerhalb der ersten 8 Wochen, innerhalb der ersten 12 Wochen, innerhalb der ersten 16 Wochen, mehr als 16 Wochen, weiß ich nicht
Hat Ihr Hund eine spezielle Furcht oder Phobie? Wenn ja, welche?<br>(Mehrfachantwort möglich)	nein, Geräusche, Gewitter/Sturm, Sonstiges:
Wie alt war Ihr Hund als Sie ihn das erste Mal alleine gelassen haben?	innerhalb der ersten 6 Wochen, innerhalb der ersten 12 Wochen, innerhalb der ersten 4 Monate, innerhalb der ersten 6 Monate, innerhalb des ersten Jahres, nach dem ersten Jahr, weiß ich nicht
Wie wurde der Hund an das Alleinebleiben gewöhnt?	keine Gewöhnung, schrittweise Gewöhnung
Wie lange gehen Sie mit Ihrem Hund insgesamt täglich Gassi (in Minuten)?<br>(Bitte keine Zeitspannen, sondern einen Durchschnittswert angeben)	frei
Wie viele Stunden verbringen Sie durchschnittlich täglich in der Gegenwart Ihres Hundes (aktiv, passiv, Tag und Nacht)?<br>(Bitte keine Zeitspannen, sondern einen Durchschnittswert angeben)	frei

Table S3. Descriptive statistics of behaviors in total of time.

Variable	N	Unit	Median	Q1	Q3	Mean	STD	Min	Max
Lying resting	17	Percent	42.73	30.63	71.52	46.87	27.54	3.76	90.00

Lying alert	17	Percent	21.48	15.39	38.13	27.29	15.55	4.20	55.00
Attention towards owner	17	Percent	18.80	8.40	25.63	18.81	12.76	0.00	44.55
Standing	17	Percent	10.00	4.03	16.54	11.40	7.76	0.62	27.15
Walking	17	Percent	5.04	2.65	9.38	10.77	13.90	1.40	50.00
Owner physical contact	17	Percent	3.36	0.63	7.05	4.67	4.70	0.00	14.57
Sitting	17	Percent	0.00	0.00	4.38	3.66	7.54	0.00	28.57
Grooming	17	Percent	0.83	0.00	4.64	3.28	5.55	0.00	21.43
Chewing	17	Percent	0.00	0.00	3.85	1.84	2.60	0.00	6.77
Panting	17	Percent	0.00	0.00	0.00	1.84	4.82	0.00	15.00
Physical contact	17	Percent	0.62	0.00	2.69	1.75	2.77	0.00	9.09
Exploring	17	Percent	0.00	0.00	0.00	1.68	4.74	0.00	14.29
Owner verbal contact	17	Percent	0.64	0.00	1.50	1.49	2.28	0.00	7.95
Following owner	17	Percent	1.32	0.66	1.92	1.47	1.38	0.00	5.71
Tail wagging	17	Percent	0.66	0.00	2.01	1.35	1.66	0.00	5.00
Attention towards sth.	17	Percent	0.00	0.00	0.00	0.83	2.25	0.00	7.50
Body shaking	17	Percent	0.00	0.00	0.83	0.47	0.59	0.00	1.68
Yawning	17	Percent	0.00	0.00	0.67	0.36	0.53	0.00	1.99
Body stretching	17	Percent	0.00	0.00	0.67	0.35	0.55	0.00	1.82
Whining	17	Percent	0.00	0.00	0.00	0.18	0.53	0.00	1.88
Owner seeks contact	17	Percent	0.00	0.00	0.00	0.12	0.27	0.00	0.75
Running	17	Percent	0.00	0.00	0.00	0.00	0.00	0.00	0.00
Play	17	Percent	0.00	0.00	0.00	0.00	0.00	0.00	0.00
Barking	17	Percent	0.00	0.00	0.00	0.00	0.00	0.00	0.00
Growling	17	Percent	0.00	0.00	0.00	0.00	0.00	0.00	0.00
Howling	17	Percent	0.00	0.00	0.00	0.00	0.00	0.00	0.00
Lip licking	17	Percent	0.00	0.00	0.00	0.00	0.00	0.00	0.00
Inviting play	17	Percent	0.00	0.00	0.00	0.00	0.00	0.00	0.00
Owner invites play	17	Percent	0.00	0.00	0.00	0.00	0.00	0.00	0.00

Table S4. Descriptive statistics of behavior groups in total of time.

Variable	N	Unit	Median	Q1	Q3	Mean	STD	Min	Max
Attentive behaviour	17	Percent	55.32	35.71	64.17	50.74	23.03	12.50	90.38
Interaction initiated by dog	17	Percent	24.06	10.08	28.75	22.04	13.46	0.00	48.18
Physically active	17	Percent	15.38	6.72	22.52	20.56	18.78	2.10	71.43
Restlessness	17	Percent	13.64	11.25	21.15	16.87	8.85	5.62	42.86
Interaction initiated by owner	17	Percent	4.55	0.63	8.33	6.28	6.53	0.00	21.19
Stress behaviour	17	Percent	3.36	1.33	5.71	4.56	4.60	0.00	15.83
Vocalising	17	Percent	0.00	0.00	0.00	0.18	0.53	0.00	1.88

Table S5. Comparison of behaviors of different separation durations.

	Sep. class 1		Sep. class 2		Sep. class 3			
**Variable**	**Median**	**IQR**	**Median**	**IQR**	**Median**	**IQR**	χ^2	**P-value**
**PeS**								
Lying alert	85.00	70.71	15.00	55.00	32.50	58.91	5.20	0.074
Lying resting	15.00	22.58	78.26	50.00	29.15	46.67	6.50	0.039
Standing	0.00	5.56	0.00	13.04	15.00	23.13	1.40	0.497
Walking	0.00	3.03	0.00	0.00	5.78	8.78	2.00	0.368

Attention towards sth.	0.00	0.00	0.00	0.00	0.00	0.00	2.00	0.368
Grooming	0.00	0.00	0.00	0.00	0.00	0.00	2.00	0.368
Chewing	0.00	5.56	0.00	0.00	0.00	16.88	4.00	0.135
Panting	0.00	0.00	0.00	0.00	0.00	0.00	2.00	0.368
Whining	0.00	0.00	0.00	0.00	0.00	0.00	2.00	0.368
Yawning	0.00	0.00	0.00	0.00	0.00	0.00	2.00	0.368
Body stretching	0.00	0.00	0.00	0.00	0.00	0.00	2.00	0.368
Following owner	0.00	2.78	0.00	0.00	1.61	7.06	2.60	0.273
Attention towards owner	77.42	75.91	11.54	6.30	46.33	17.46	2.80	0.247
Owner physical contact	0.00	2.50	0.00	3.85	0.00	0.00	2.60	0.273
Owner verbal contact	0.00	2.78	0.00	0.00	0.00	0.00	2.00	0.368
Owner seeks contact	0.00	0.00	0.00	0.00	0.00	0.00	2.00	0.368
Restlessness	8.33	6.59	5.00	2.50	12.92	15.48	2.21	0.331
Physically active	6.06	13.89	0.00	11.54	23.33	27.53	6.00	0.050
Attentive behaviour	105.00	76.45	30.00	74.46	83.75	68.17	3.60	0.165
Interaction by dog	77.42	72.88	11.54	6.30	47.94	17.10	2.80	0.247
Interaction by owner	2.50	5.56	0.00	3.85	0.00	3.75	1.27	0.529
Vocalising	0.00	0.00	0.00	0.00	0.00	0.00	2.00	0.368
Stress behaviour	0.00	2.50	0.00	0.00	0.00	1.88	0.50	0.779
**E1**								
Lying alert	12.50	36.25	0.00	10.00	10.00	5.00	1.00	0.607
Lying resting	17.50	83.75	95.00	25.00	60.00	45.71	3.45	0.178
Sitting	0.00	0.00	0.00	0.00	0.00	0.00	2.00	0.368
Standing	10.00	20.00	0.00	5.00	15.00	5.00	4.43	0.109
Walking	2.50	42.50	0.00	10.00	10.00	10.00	6.86	0.032
Grooming	0.00	10.71	0.00	5.00	0.00	10.00	0.80	0.670
Chewing	0.00	0.00	0.00	0.00	0.00	10.00	4.00	0.135
Panting	0.00	0.00	0.00	0.00	0.00	0.00	2.00	0.368
Body stretching	0.00	0.00	0.00	0.00	0.00	10.00	2.00	0.368
Attention towards sth.	0.00	0.00	0.00	0.00	0.00	15.00	4.00	0.135
Restlessness	9.64	24.82	5.00	25.00	25.00	5.00	2.80	0.247
Physically active	27.14	81.43	0.00	25.00	10.00	25.00	5.20	0.074
Attentive behaviour	12.50	36.25	0.00	10.00	25.00	10.00	1.00	0.607
Stress behaviour	0.00	10.71	0.00	0.00	0.00	0.00	3.71	0.156
**E2**								
Lying alert	15.00	23.33	5.00	5.00	10.00	5.89	1.27	0.529
Lying resting	65.00	53.89	95.00	30.00	80.36	32.68	5.57	0.062
Standing	11.11	30.00	0.00	10.00	3.57	13.04	2.60	0.273
Walking	5.00	22.22	0.00	10.00	0.00	7.50	2.00	0.368
Attention towards sth.	0.00	0.00	0.00	0.00	0.00	0.00	2.00	0.368
Grooming	0.00	33.33	0.00	0.00	0.00	0.00	2.00	0.368
Panting	0.00	0.00	0.00	0.00	0.00	0.00	2.00	0.368
Yawning	0.00	0.00	0.00	0.00	0.00	0.00	2.00	0.368
Restlessness	10.00	25.00	5.00	20.00	15.71	29.11	2.60	0.273
Physically active	35.00	50.56	5.00	10.00	5.00	21.25	4.91	0.086
Attentive behaviour	25.00	20.00	5.00	5.00	15.00	15.89	1.73	0.420
Stress behaviour	0.00	0.00	0.00	0.00	0.00	0.00	2.00	0.368
**L1**								
Lying alert	0.00	0.00	0.00	30.00	0.00	10.00	3.00	0.223
Lying resting	100.00	0.00	70.00	50.00	5.00	100.00	3.80	0.150
Standing	0.00	0.00	0.00	0.00	0.00	0.00	1.00	0.607
Walking	0.00	0.00	0.00	0.00	0.00	0.00	1.00	0.607

Grooming	0.00	0.00	0.00	0.00	0.00	0.00	2.00	0.368
Panting	0.00	0.00	0.00	0.00	0.00	0.00	2.00	0.368
Yawning	0.00	0.00	0.00	0.00	0.00	0.00	2.00	0.368
Body stretching	0.00	0.00	0.00	5.00	0.00	0.00	4.00	0.135
Body shaking	0.00	0.00	0.00	5.00	0.00	0.00	4.00	0.135
Restlessness	5.00	5.00	10.00	10.00	5.00	5.00	0.93	0.627
Physically active	0.00	0.00	0.00	0.00	0.00	40.00	3.71	0.156
Attentive behaviour	0.00	0.00	0.00	30.00	0.00	10.00	3.00	0.223
Stress behaviour	0.00	0.00	0.00	15.00	0.00	0.00	2.00	0.368
**L2**								
Lying alert	25.00	30.00	35.00	40.00	45.00	60.00	0.43	0.807
Lying resting	65.00	20.00	65.00	70.00	55.00	80.00	1.08	0.584
Standing	0.00	0.00	0.00	0.00	0.00	10.00	2.00	0.368
Walking	0.00	0.00	0.00	10.00	0.00	0.00	3.00	0.223
Attention towards sth.	0.00	0.00	0.00	0.00	0.00	0.00	2.00	0.368
Grooming	0.00	0.00	5.00	25.00	0.00	0.00	4.00	0.135
Tail wagging	0.00	0.00	0.00	0.00	0.00	0.00	2.00	0.368
Body stretching	0.00	0.00	0.00	0.00	0.00	0.00	4.00	0.135
Restlessness	5.00	5.00	5.00	5.00	5.00	15.00	2.71	0.257
Physically active	0.00	0.00	10.00	30.00	0.00	10.00	5.00	0.082
Attentive behaviour	35.00	15.00	35.00	45.00	45.00	60.00	0.43	0.807
Stress behaviour	0.00	0.00	0.00	0.00	0.00	0.00	2.00	0.368
**PoS**								
Lying alert	32.50	11.67	10.00	35.00	15.00	4.40	2.80	0.247
Lying resting	15.00	20.00	46.15	62.50	8.85	62.45	4.00	0.135
Sitting	0.00	17.50	0.00	0.00	0.00	0.00	2.00	0.368
Standing	39.13	25.00	14.29	20.58	24.01	25.88	3.26	0.196
Walking	10.00	9.89	22.50	23.27	21.59	31.50	3.11	0.211
Grooming	0.00	0.00	0.00	0.00	0.00	0.00	2.00	0.368
Chewing	2.50	10.00	0.00	0.00	0.00	1.88	3.80	0.150
Panting	0.00	0.00	0.00	0.00	0.00	0.00	2.00	0.368
Tail wagging	2.50	8.70	0.00	5.00	6.42	11.34	0.67	0.717
Whining	0.00	0.00	0.00	0.00	0.00	0.00	2.00	0.368
Yawning	2.50	3.33	0.00	0.00	0.00	0.00	2.92	0.232
Body stretching	0.00	0.00	0.00	0.00	0.00	0.00	2.00	0.368
Body shaking	2.50	4.35	0.00	7.14	0.00	0.00	2.92	0.232
Following owner	5.00	2.50	7.14	10.00	5.95	12.01	0.93	0.627
Physical contact	0.00	4.35	2.50	7.14	8.15	14.07	1.73	0.420
Attention towards owner	60.00	7.50	50.00	44.42	32.23	38.50	0.95	0.623
Owner physical contact	25.00	33.62	7.69	11.79	18.62	35.31	5.20	0.074
Owner verbal contact	0.00	4.35	7.14	7.50	3.45	7.35	0.55	0.761
Owner seeks contact	0.00	2.50	0.00	0.00	0.00	0.00	4.00	0.135
Restlessness	30.00	10.00	22.50	22.86	36.29	28.25	0.74	0.692
Physically active	30.00	22.50	37.50	31.15	49.31	47.06	1.20	0.549
Attentive behaviour	95.00	28.04	82.50	23.08	78.46	65.05	2.80	0.247
Interaction by dog	70.00	22.50	65.00	28.02	70.96	23.93	0.74	0.692
Interaction by owner	27.50	29.28	21.43	35.96	22.07	47.19	1.20	0.549
Vocalising	0.00	0.00	0.00	0.00	0.00	0.00	2.00	0.368
Stress behaviour	7.50	9.71	12.50	7.31	9.40	13.45	0.11	0.946

Table S6. Effect sizes and p-values on SRB status.

Variable	Effect size	Strength	P-value	Method
General information				
Gender	0.017	negligible	0.631	Chi2 & CramersV
Status	0.041	negligible	0.580	Fisher & CramersV
Age	0.008	negligible	0.820	MWU & R
Breed	0.097	negligible	0.005**	Chi2 & CramersV
Height	0.204	small	0.080	Welch & CohensD
Weight	0.253	small	0.021*	Welch & CohensD
Background	0.131	small	0.017*	Fisher & CramersV
Shelter	0.111	small	0.001**	Chi2 & CramersV
Time in shelter	0.167	small	0.012*	MWU & R
Time with owner	0.090	negligible	0.008**	MWU & R
Number of owners	0.033	negligible	0.344	MWU & R
Adoption age	0.004	negligible	0.900	MWU & R
Gender owner	0.016	negligible	1	Fisher & CramersV
Age owner	0.086	negligible	0.011*	MWU & R
Men in household	0.090	negligible	0.513	Welch & CohensD
Women in household	0.022	negligible	0.833	Welch & CohensD
Children in household	0.122	negligible	0.331	Welch & CohensD
Living environment	0.000	negligible	1	Chi2 & CramersV
Garden	0.020	negligible	0.547	Chi2 & CramersV
Other pet	0.077	negligible	0.023*	Chi2 & CramersV
Dog	0.001	negligible	1	Fisher & CramersV
Cat	0.069	negligible	0.045*	Chi2 & CramersV
Rodent	0.048	negligible	0.158	Chi2 & CramersV
Bird	0.020	negligible	0.558	Chi2 & CramersV
Horse & Cattle	0.078	negligible	0.019*	Fisher & CramersV
Sheep & Goat	0.000	negligible	0.532	Fisher & CramersV
Fish	0.000	negligible	1	Fisher & CramersV
Reptiles	0.000	negligible	0.675	Fisher & CramersV
Changes	0.051	negligible	0.135	Chi2 & CramersV
Working hours	0.020	negligible	0.555	Chi2 & CramersV
Moving	0.016	negligible	0.643	Chi2 & CramersV
New family member	0.048	negligible	0.153	Chi2 & CramersV
Loss family member	0.022	negligible	0.511	Chi2 & CramersV
Health	0.002	negligible	0.646	Fisher & CramersV
Less activity	0.017	negligible	0.617	Chi2 & CramersV
More activity	0.000	negligible	1	Fisher & CramersV
New owner	0.005	negligible	0.634	Fisher & CramersV
Other change	0.051	negligible	0.077	Fisher & CramersV
Separation information				
Separation frequency	0.287	small	0.009**	Welch & CohensD
Separation period	0.134	small	< .001***	MWU & R
Maximum separation	0.111	small	< .001***	MWU & R
Walking time prior	0.082	negligible	0.434	Welch & CohensD
Time between walk & separation	0.031	negligible	0.358	MWU & R
Leaving				
Leaving ritual	0.071	negligible	0.036*	Chi2 & CramersV
**Variable**	**Effect size**	**Strength**	**P-value**	**Method**

Type of leaving ritual	0.092	negligible	0.071	Fisher & CramersV
Intensity leaving ritual	0.012	negligible	0.722	MWU & R
Worries owner during separation	0.216	small	< .001***	MWU & R
Worries dog during separation	0.384	medium	< .001***	MWU & R
Separation location				
Inside	0.099	negligible	0.007**	Fisher & CramersV
Outside	0.008	negligible	0.809	Chi2 & CramersV
Property	0.023	negligible	0.492	Chi2 & CramersV
Outside kennel	0.019	negligible	0.611	Fisher & CramersV
Inside kennel	0.082	negligible	0.014*	Fisher & CramersV
One room	0.106	small	0.002**	Chi2 & CramersV
Several rooms	0.160	small	< .001**	Chi2 & CramersV
Exercise during separation	0.135	small	0.093	Chi2 & CramersV
Toy	0.116	small	< .001***	Chi2 & CramersV
Food	0.123	small	< .001***	Chi2 & CramersV
Other pet during separation	0.037	negligible	0.277	Chi2 & CramersV
Dog	0.008	negligible	0.808	Chi2 & CramersV
Cat	0.062	negligible	0.025*	Chi2 & CramersV
Bird	0.000	negligible	1	Fisher & CramersV
Reptile	0.045	negligible	0.102	Fisher & CramersV
Rodent	0.000	negligible	1	Fisher & CramersV
Symptoms				
Whining	0.426	medium	< .001***	MWU & R
Barking	0.350	medium	< .001***	MWU & R
Howling	0.363	medium	< .001***	MWU & R
Urination	0.077	negligible	0.024*	MWU & R
Defecation	0.067	negligible	0.048*	MWU & R
Destruction	0.174	small	< .001***	MWU & R
Salivation	0.328	medium	< .001***	MWU & R
Shaking	0.297	small	< .001***	MWU & R
Restlessness	0.486	medium	< .001***	MWU & R
Growling	0.121	small	< .001***	MWU & R
Excitement	0.473	medium	< .001***	MWU & R
Lip licking	0.357	medium	< .001***	MWU & R
Yawning	0.261	small	< .001***	MWU & R
Escape behaviour	0.280	small	< .001***	MWU & R
Greeting				
Greeting ritual	0.107	small	0.002**	Chi2 & CramersV
Type of ritual	0.121	small	0.005**	Chi2 & CramersV
Intensity greeting owner	0.083	negligible	0.014*	MWU & R
Intensity greeting dog short	0.164	small	< .001***	MWU & R
Intensity greeting dog long	0.192	small	< .001***	MWU & R
Time to relaxation	0.936	large	< .001***	Welch & CohensD
Habituation	0.036	negligible	0.287	Chi2 & CramersV
Daily walking	0.082	negligible	0.393	Welch & CohensD
Presence of owner	0.236	small	0.046*	Welch & CohensD
Separation mother	0.055	negligible	0.162	MWU & R
Age first separation from owner	0.034	negligible	0.336	MWU & R
Phobia	0.014	negligible	0.675	Chi2 & CramersV
**Variable**	**Effect size**	**Strength**	**P-value**	**Method**
Thunderstorm	0.000	negligible	0.989	Chi2 & CramersV

Noise	0.015	negligible	0.657	Chi2 & CramersV
Humans	0.055	negligible	0.071	Fisher & CramersV
Dogs	0.001	negligible	1	Fisher & CramersV
Objects	0.022	negligible	0.500	Fisher & CramersV
Environments	0.027	negligible	0.193	Fisher & CramersV
Vehicles	0.012	negligible	0.711	Fisher & CramersV
Surfaces	0.000	negligible	0.478	Fisher & CramersV
Separation	0.078	negligible	0.029*	Fisher & CramersV
Others	0.065	negligible	0.037*	Fisher & CramersV
Attachment				
People substituting	0.107	negligible	0.374	Welch & CohensD
Animal welfare	0.118	negligible	0.300	Welch & CohensD
General attachment	0.155	negligible	0.194	Welch & CohensD
Total attachment	0.003	negligible	0.981	Welch & CohensD
Personality				
Trainabaility	0.180	negligible	0.130	Welch & CohensD
Boldness	0.026	negligible	0.834	Welch & CohensD
Sociability	0.026	negligible	0.937	Welch & CohensD
Calmness	0.723	medium	< .001***	Welch & CohensD
Emotional disposition				
Pessimism	0.672	medium	< .001***	Welch & CohensD
Pos. Activation	0.654	medium	< .001***	Welch & CohensD
Interest	0.122	negligible	0.296	Welch & CohensD
Excitability	0.788	medium	< .001***	Welch & CohensD
Persistence	0.612	medium	< .001***	Welch & CohensD
